# Harnessing nucleotide metabolism to control glycosylase base editing outcomes

**DOI:** 10.7150/thno.125705

**Published:** 2026-02-04

**Authors:** Rui Tao, Min Li, Junyi Fei, Minhai Tang, Zhi Yang, Yun Hu, Yaoge Jiao, Zhangxue Hu, Shaohua Yao

**Affiliations:** 1 Institute of Kidney Diseases, West China Hospital, Sichuan University, Chengdu 610041, Sichuan, China.; 2 Laboratory of Biotherapy, National Key Laboratory of Biotherapy, Cancer Center, West China Hospital, Sichuan university, Chengdu 610041, Sichuan, China.; 3 Department of Nephrology, West China Hospital, Sichuan University, Chengdu, 610041, Sichuan, China.; 4 Tianfu Jincheng Laboratory, Chengdu 610095, Sichuan, China.

**Keywords:** base editing, nucleotide metabolism, glycosylase-derived base editor, translesion synthesis, transversion

## Abstract

Rationale: Glycosylase-derived base editors enable transversion base substitutions, expanding the scope of genome engineering for both basic research and clinical applications. However, the variable outcomes and low efficiency of B (C/G/T)-to-A editing in mammalian cells hinder their broader utility, likely due to inefficient thymine translesion synthesis (TLS) across apurinic/apyrimidinic (AP) sites.

Methods and Results: We developed a nucleotide metabolism-based strategy to enhance B-to-A editing by leveraging endogenous nucleotide metabolism. We showed that elevating intracellular deoxythymidine triphosphate (dTTP) levels via exogenous thymidine (dT) supplementation, which activates the thymidine kinase 1 (TK1)-dependent salvage pathway for the production of dTTP, increased C-to-A, G-to-A, and T-to-A editing efficiencies by up to 4-fold, 1.8-fold, and 1.8-fold, respectively, and improved A-product purity by up to 2.7-fold. Moreover, supplementation with dA increased T outcomes, albeit at a relatively modest level. In a disease-relevant single nucleotide variation (SNV) model, dT treatment enabled efficient generation of pathogenic mutations otherwise inaccessible to base editing.

Conclusion: Our findings establish metabolic modulation as a powerful means to control base editing outcomes and expand the functional capabilities of glycosylase-derived editors.

## Introduction

Base editing has emerged as a transformative genome editing approach, enabling precise single-nucleotide substitutions without introducing DNA double-strand breaks (DSBs). The first generation of base editors employed cytidine or adenine deaminases to induce precise C-to-T and A-to-G conversions 1, 2. More recently, glycosylase-based editors such as CGBE, gGBE, AYBE, and TBE have extended the editing scope by leveraging endogenous or engineered glycosylases to induce diverse base transversions (e.g., C-to-G, G-to-C, A-to-C, T-to-G) 3-12. These advances significantly broaden the versatility of base editing, creating new opportunities in gene therapy, functional genomics, and synthetic biology.

Glycosylase-based base editors operate by creating apurinic/apyrimidinic (AP) sites through glycosylase-mediated base excision within the nCas9-targeted site 13, 14. The resulting AP sites are then repaired by endogenous DNA repair pathways, among which translesion synthesis (TLS) pathway is considered critical for base conversion 15-17. Error-prone TLS polymerases such as Pol η, Pol κ, and REV1 can be recruited to insert non-template-dependent bases against the AP sites, leading to nucleotide transversions (e.g., C-to-G, A-to-T) 15, 18-22. Despite their promising potential, current glycosylase base editors are limited by low efficiency and product purity. In particular, CGBE-, gGBE-, and TBE-mediated B(C/G/T)-to-A conversions remain inefficient 3, 5, 7, 9-12, restricting their utility in applications requiring robust introduction of A•T base pairs, such as creating stop codons, correcting splicing mutations, or modeling single-nucleotide variants.

The TLS polymerases in eukaryotic cells display relatively weak substrate specificity, except for REV1, which specifically inserts cytosine against AP site 20, 23. For example, Pol η can incorporate all four deoxynucleotides in vitro, with a preference for dATP and dTTP over dGTP and dCTP 23. Pol κ and Pol ι also display low selectivity, though with distinct tendencies and specificities. Pol κ incorporates nucleotides with low fidelity but demonstrates a moderate preference for dCTP and dATP, whereas Pol ι exhibits a strong preference for incorporating dGTP and dTTP 23, 24. Such promiscuous features of TLS enzymes and their functional redundancy might explain why glycosylase-based base editors tend to generate multiple outcomes in the target sites. Notably, these substrate preferences are typically observed under conditions with pure deoxynucleotide substrates, suggesting that for polymerases with low intrinsic specificity, altering nucleotide availability may shift their insertion bias toward the most abundant dNTP. Therefore, we hypothesize that modulating the intracellular concentration or ratio of specific dNTPs could influence the editing outcomes of glycosylase-based base editors.

To test this hypothesis, we evaluated whether increasing specific dNTP levels through deoxynucleoside supplementation could influence glycosylase-based base editing. Using a C-to-A reporter system as well as endogenous genomic targets, we observed that thymidine (dT) supplementation markedly enhanced C-to-A editing by CGBE, G-to-A editing by gGBE, and T-to-A editing by DAF-TBE. We found that this enhancement did not result from cell-cycle arrest, because treatment with other compounds that similarly induce S-phase blockade failed to affect editing outcomes. Instead, the effect was dependent on dTTP production, since knockdown of *TK1*, the rate-limiting enzyme in the dT salvage pathway, abolished the dT-dependent increase in editing efficiency. Similarly, dA or dG supplementation increased T or C editing outcomes respectively. Together, these results established nucleotide metabolic modulation as an effective strategy to control glycosylase base editor outcomes, thereby expanding the functional versatility of these editors, and suggested a possible mechanism of point mutations induced by AP sites generated through the excision of damaged bases by endogenous glycosylase.

## Results

### Y66D fluorescent reporter validates dT-mediated optimization of C-to-A conversion

To investigate the effect of dTTP levels on glycosylase-based B(C/G/T)-to-A editing efficiency, we first tested whether exogenous thymidine (dT) treatment could elevate intracellular dTTP in HEK293T cells. Using LC-MS, we found that a 24 h treatment with a range of dT concentrations (500 μM-5 mM) increased dTTP levels in a dose-dependent manner, with a 0.6- to 9.2-fold increase (Figure S1).

**Figure 1:**
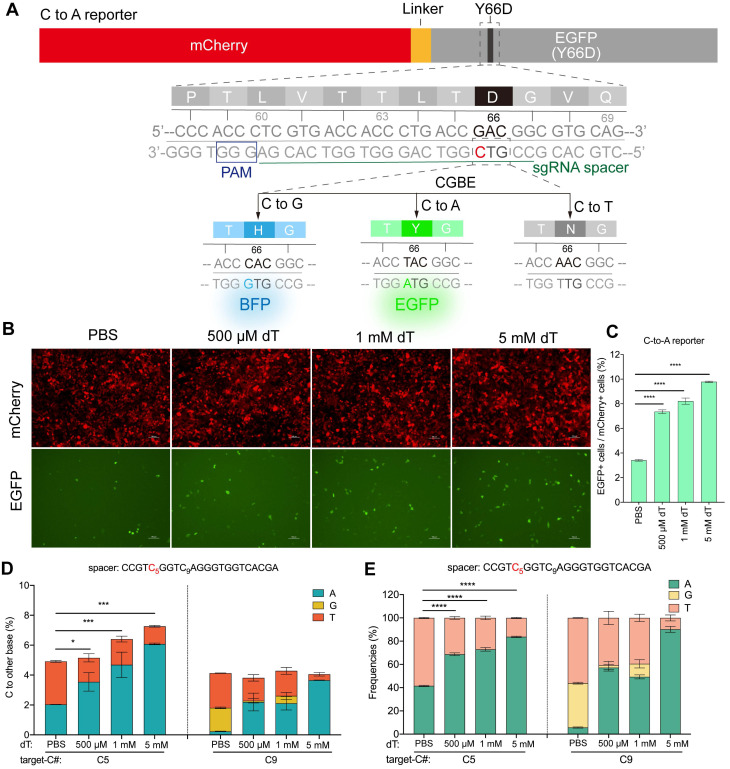
** Thymidine (dT) enhances CGBE-mediated C-to-A editing in fluorescent reporter system. A.** Schematic of the C-to-A specific reporter design. PAM: protospacer adjacent motif. **B.** Fluorescence images of PBS control versus dT (500 μM/1 mM/5 mM) treatments showing dose-dependent response. Scale bar: 100 μm. **C.** Percentage of EGFP-positive cells after treatment with PBS or a gradient of dT concentrations. **D.** C-to-D (A/G/T) conversion frequencies at EGFP Y66D locus quantified by targeted sequencing.** E.** Editing product distributions comparing PBS with dT. The presented data are representative of three independent experiments, and error bars represent the standard deviation of the mean (* *p* < 0.05, *** *p* < 0.001, **** *p* < 0.0001).

Next, we assessed the effect of dT supplementation on CGBE editing outcomes. We first developed a C-to-A responsive fluorescent reporter system (Figure 1A). This system expresses mCherry as an internal control and a disrupted EGFP as a reporter. In the EGFP reporter, the Y66 codon (TAC) located in the chromophore of GFP was mutated to D66 (GAC), which abolishes green fluorescence. Only C-to-A editing at this site restores the wild-type TAC codon (corresponding to a G-to-T edit on the antisense strand), thereby reactivating green fluorescence, whereas C-to-G or C-to-T edits produce blue fluorescence or inactivate EGFP, respectively 25 (Figure S2). Therefore, this design allows rapid, flow cytometry-based quantification of C-to-A editing efficiency (Figure S3).

With this reporter, we found that dT treatment significantly increased the proportion of EGFP-positive cells in a dose-dependent manner. Compared with the PBS control, the percentage of EGFP-positive cells increased by 1.2-, 1.4-, and 1.9-fold in the 500 μM, 1 mM, and 5 mM dT groups, respectively (Figure 1B-C). Next-generation sequencing confirmed that dT enhanced CGBE-mediated C-to-A editing in a dose-dependent fashion: editing efficiency at the target C5 site increased from 2.0% in PBS-treated cells to 3.6%, 4.7%, and 6.1% with 500 μM, 1 mM, and 5 mM dT, respectively (Figure 1D). Editing product purity also improved, with the proportion of A among edited products rising from 41.5% in the PBS group to 68.7%, 73.0%, and 83.8% in the 500 μM, 1 mM, and 5 mM dT groups, respectively (Figure 1E). Low-level editing was also observed at the neighboring C9 site, resulting in a synonymous mutation, with efficiency and purity trends similar to those at C5 (Figure 1D-E).

To further examine whether higher concentrations of dT could elevate intracellular dTTP levels and thereby further enhance C-to-A editing, we tested a broader range of dT concentrations (8-50 mM). Intracellular dTTP levels increased in a concentration-dependent manner and reached a maximum at 20 mM dT, followed by a decline at 30 mM (Figure S4A). In parallel, cell viability decreased significantly at dT concentrations above 8 mM (*p* < 0.05), reaching approximately 60% of control levels at 20 mM (Figure S4B). These findings suggest that high dT concentrations may indirectly limit further accumulation of dTTP by compromising cell viability. Consistent with this notion, in the EGFP reporter assay, both the efficiency and purity of C-to-A editing plateaued at 5 mM dT and did not further increase despite higher intracellular dTTP levels at elevated dT concentrations (Figure S4C-E). This apparent plateau is likely attributable, at least in part, to reduced cell viability at higher dT concentrations. Collectively, these results demonstrated that dT supplementation enhances both efficiency and product purity of CGBE-mediated C-to-A editing.

### dT supplement generally enhanced the B(C/G/T)-to-A editing activity of various glycosylase-derived editors

**Figure 2:**
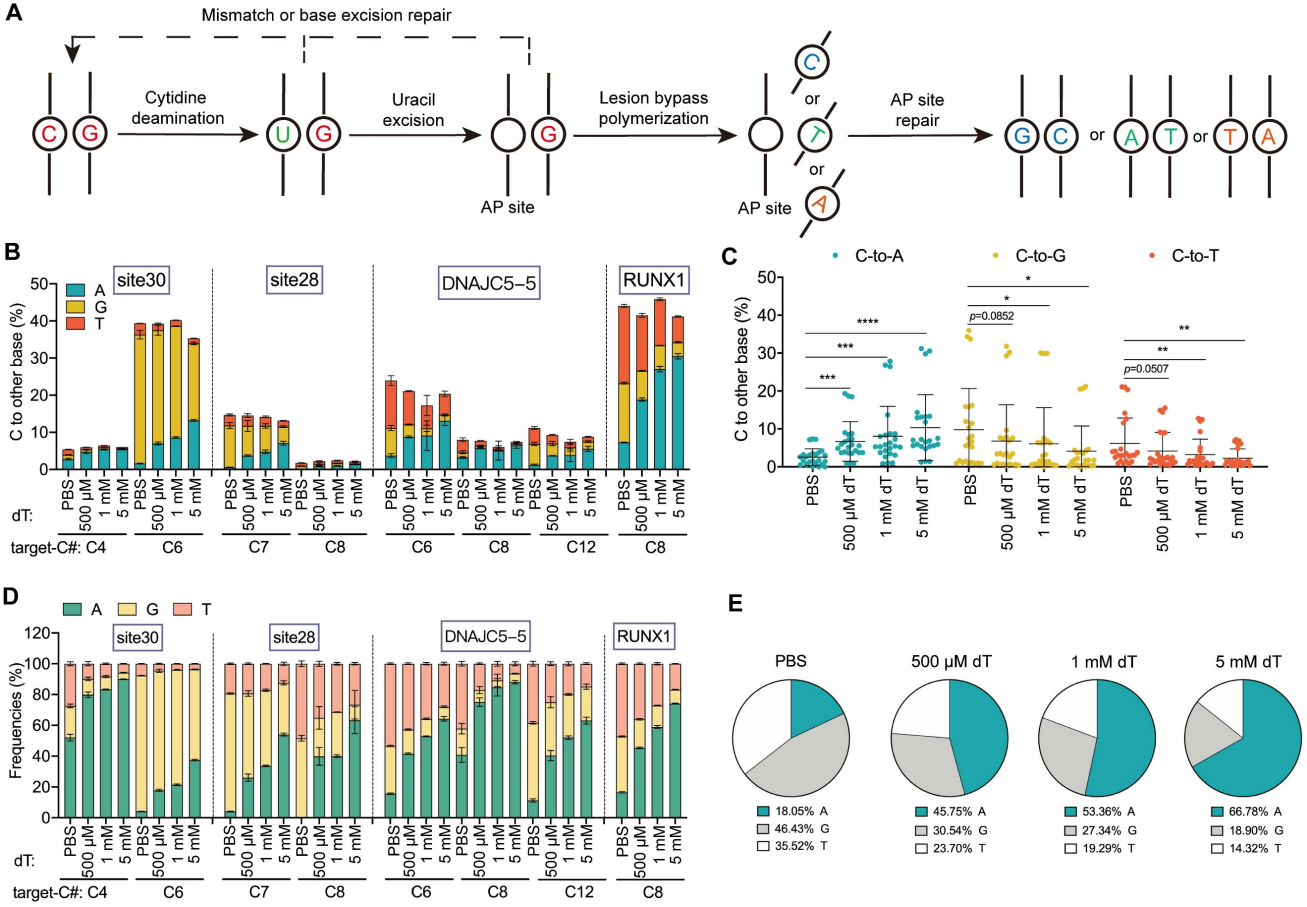
** dT enhances C-to-A conversions in CGBE. A.** Schematic diagram illustrating the potential editing pathways and outcomes following CGBE-induced cytosine deamination. **B.** Endogenous locus editing efficiencies of CGBE with graded dT concentrations in HEK293T cells. The presented data are representative of three independent experiments, and error bars represent the standard deviation of the mean. **C.** The C-to-A, C-to-G, and C-to-T editing efficiencies across all replicates and target sites. Data for each editing outcome are pooled from all four endogenous loci and three biological replicates (* *p* < 0.05, ** *p* < 0.01, *** *p* < 0.001). **D.** Product distribution of CGBE-mediated editing with different dT concentrations. The presented data are representative of three independent experiments, and error bars represent the standard deviation of the mean. **E.** Aggregate proportions of all editing outcomes across the four tested sites.

We then determine the effect of dT supplementation on the outcomes of CGBE at endogenous loci (Figure 2A) 12. We found that treatment with 500 μM, 1 mM, and 5 mM dT led to a dose-dependent increase in average C-to-A editing efficiency, reaching 6.7%, 8.1%, and 10.4%, respectively, compared to 2.6% in the PBS control. This corresponds to a 1.6-4.0-fold increase (Figure 2B-C). Among the tested loci, *RUNX1* showed the most significant response to dT, with C-to-A editing efficiency increasing from 7.3% in the PBS group to 18.8%, 27.0%, and 30.5% upon treatment (Figure 2B). Notably, dT treatment dose-dependently reduced non-A editing outcomes, with C-to-G efficiency declining from 9.8% in PBS to 6.8%, 6.1%, and 4.2% with 500 μM, 1 mM, and 5 mM dT, respectively, and C-to-T efficiency decreasing from 6.2% to 4.2%, 3.3%, and 2.3% (Figure 2C). Consistently, dT markedly improved C-to-A editing purity, with average purities of 45.8%, 53.4%, and 66.8% in 500 μM, 1 mM, and 5 mM, respectively, representing 1.5-2.7-fold increases over PBS (18.1%; Figure 2D-E). To assess whether further increases in dT concentration could enhance editing outcomes, we tested concentrations up to 20 mM. However, both C-to-A editing efficiency and product purity seemed to approach saturation at dT concentrations above 5 mM, with no significant additional improvement observed at higher doses (Figure S5). This saturation behavior is consistent with the plateau observed in the EGFP reporter assay (Figure S4) and likely reflects a dynamic balance between intracellular dTTP accumulation and dT-induced cytotoxicity at elevated concentrations.

**Figure 3:**
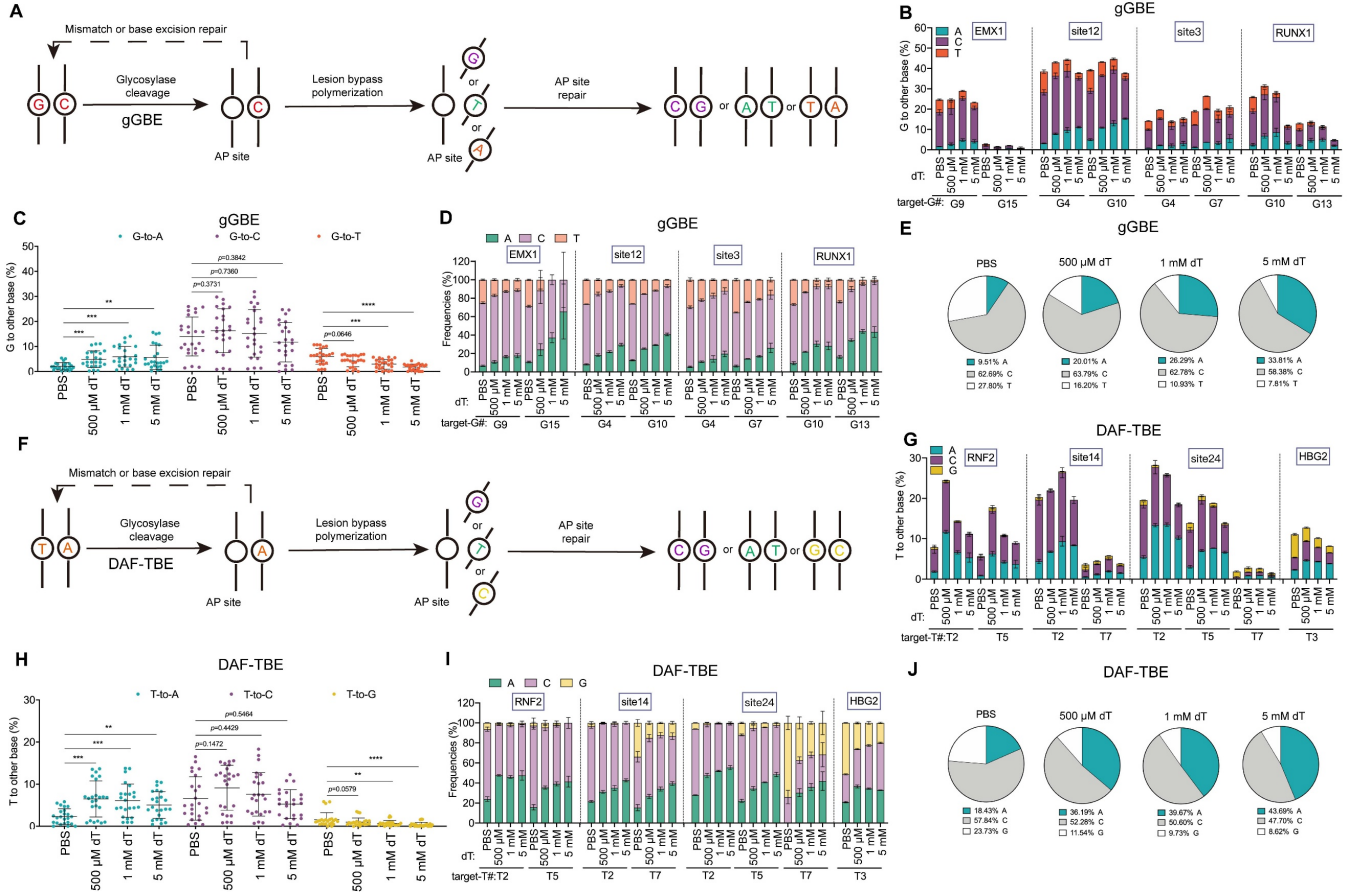
** dT enhances G-to-A and T-to-A conversions. A.** Schematic diagram illustrating the potential DNA repair pathways and outcomes initiated by gGBE-mediated excision of guanine. **B.** Endogenous locus editing efficiencies of gGBE with graded dT concentrations in HEK293T cells. **C.** The G-to-A, G-to-C, and G-to-T editing efficiencies across all replicates and target sites. (* *p* < 0.05, ** *p* < 0.01, *** *p* < 0.001, **** *p* < 0.0001). **D.** Product distribution of gGBE-mediated editing with different dT concentrations. **E.** Aggregate proportions of all editing outcomes from gGBE-mediated base editing across four tested sites. **F.** Schematic diagram illustrating the potential DNA repair pathways and outcomes initiated by DAF-TBE-mediated excision of thymine. **G.** Endogenous locus editing efficiencies of DAF-TBE with graded dT concentrations in HEK293T cells. **H.** The T-to-A, T-to-C, and T-to-G editing efficiencies across all replicates and target sites (* *p* < 0.05, ** *p* < 0.01, *** *p* < 0.001, **** *p* < 0.0001). **I.** Product distribution of DAF-TBE-mediated editing with different dT concentrations. **J.** Aggregate proportions of all editing outcomes from DAF-TBE-mediated base editing across four tested sites.

Next, to test whether the enhancement of A-editing products was specific to CGBE, we examined the effect of dT on gGBE, which functions to catalyze targeted guanine glycosylation to induce G to other bases conversion (Figure 3A) 7. Similar to observations with CGBE, dT treatment dose-dependently enhanced G-to-A editing in gGBE, albeit with some site-specific variability, increasing average efficiency from 2.1% in PBS to 4.9%, 5.9%, and 5.6% with 500 μM, 1 mM, and 5 mM, respectively (Figure 3B-C). At the same time, non-A editing products decreased, with G-to-T edits being the most sensitive to dT. For example, G-to-T editing was almost completely suppressed at G15 of the *EMX1* locus and G13 of the *RUNX1* locus (Figure 3B), while G-to-C edits were rather less sensitive to dT treatment (Figure 3C). Regarding editing purity, dT supplementation markedly improved G-to-A purity from 9.5% in PBS to 20.0%, 26.3%, and 33.8% with 500 μM, 1 mM, and 5 mM dT, respectively (Figure 3D-E), suggesting that the effect of dT to evaluate A editing outcomes is a rather general effect.

Then we tested the effect of dT on DAF-TBE base editor that catalyzes the glycosylation of normal thymine to produce T to other bases conversion (Figure 3F) 9. Consistent with observations in CGBE and gGBE, dT treatment significantly enhanced T-to-A editing efficiency in the DAF-TBE system. Treatment with 500 μM, 1 mM, and 5 mM dT increased the average T-to-A efficiency from 2.32% in PBS to 6.5%, 6.1%, and 5.1%, respectively (Figure 3G-H). Interestingly, T-to-C editing remained largely unaffected by dT, whereas T-to-G editing decreased significantly as the dT concentration increased (PBS: 1.6%; 500 μM: 1.0%; 1 mM: 0.7%; 5 mM: 0.4%) (Figure 3H). Overall, dT treatment markedly improved T-to-A editing purity, reaching 36.2%, 39.7%, and 43.7% at 500 μM, 1 mM, and 5 mM, respectively, 1.0-1.4-fold increases over PBS (18.4%; Figure 3I-J).

To further assess whether the dT-associated enhancement extends across different cellular contexts, we examined the effects of dT supplementation on B-to-A editing mediated by the three base editors described above in HeLa cells. Consistent with the results observed in HEK293T cells, dT treatment was associated with increased B-to-A editing efficiency and improved product purity in HeLa cells (Figure S6), demonstrating that dT supplementation reliably enhances B-to-A editing across different cell types.

Notably, dT treatment did not obviously affect indel frequencies in any of the tested base editing systems. For example, in the CGBE system, indel rates were comparable between dT-treated groups (500 μM: 1.1%; 1 mM: 1.1%; 5 mM: 1.4%) and PBS control (1.2%; *p* > 0.05; Figure S7). Collectively, these results demonstrate that dT supplementation broadly enhances B(C/G/T)-to-A editing efficiencies by multiple glycosylase-derived editors without increasing indel formation.

### dT enhances B(C/G/T)-to-A editing independently of cell cycle arrest

**Figure 4:**
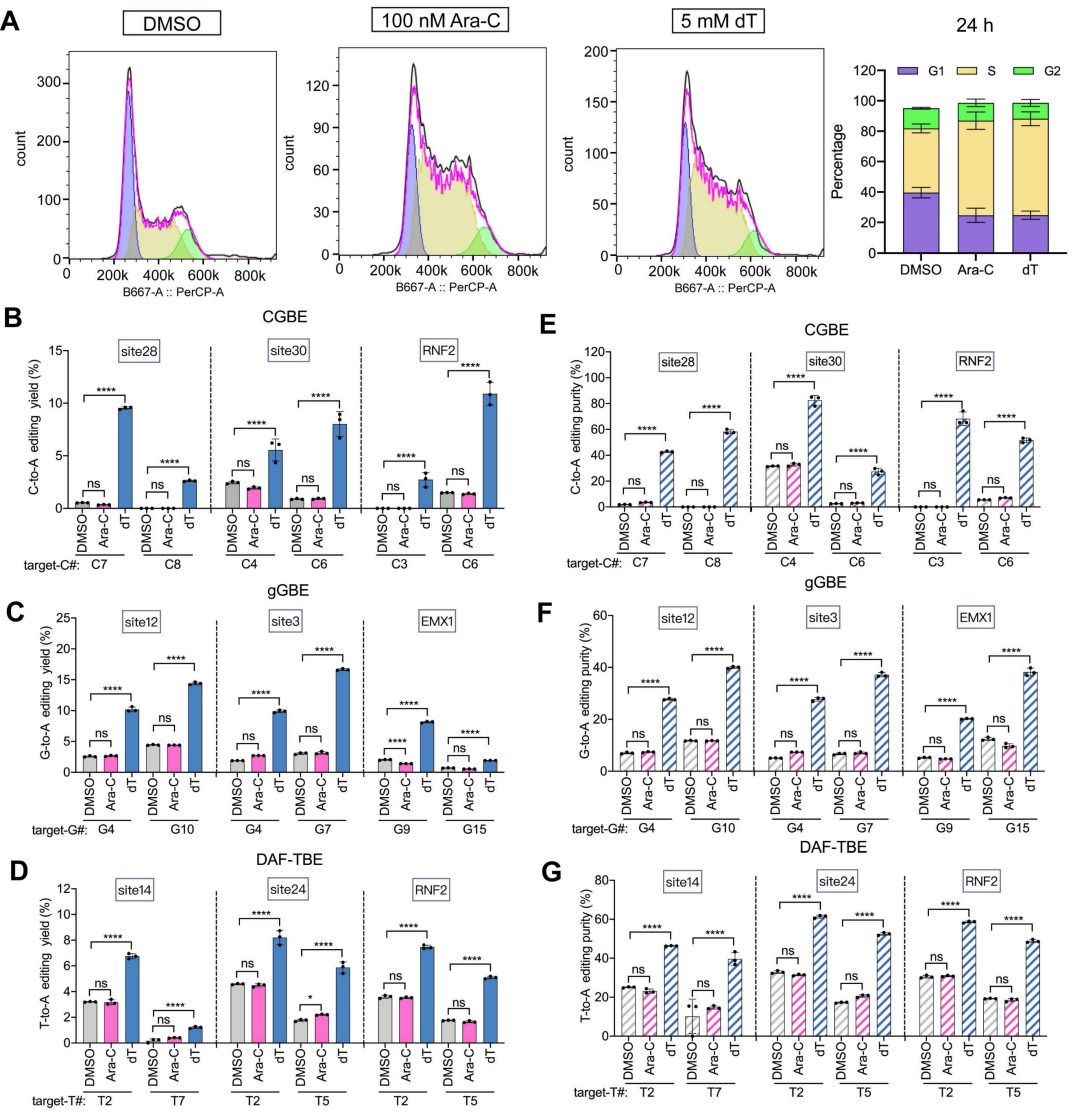
** Effects of cell cycle modulation on glycosylase base editing outcomes. A.** Cell cycle analysis following 24-hour treatment with DMSO (control), cytarabine (Ara-C, S-phase blocker), or dT (S-phase blocker). Left panels: FlowJo-modeled cell cycle distributions; right panel: quantitative phase allocation (see Methods). **B.** C-to-A editing efficiency of CGBE under DMSO, Ara-C, or dT treatment. **C.** G-to-A editing efficiency of gGBE under DMSO, Ara-C, or dT treatment.** D.** T-to-A editing efficiency of DAF-TBE under DMSO, Ara-C, or dT treatment.** E.** C-to-A product purity of CGBE-mediated editing across treatment conditions. **F.** G-to-A product purity of gGBE-mediated editing across treatment conditions. **G.** T-to-A product purity of DAF-TBE-mediated editing across treatment conditions (**** *p* < 0.0001).

Since high concentrations of thymidine can induce S-phase arrest 26, we sought to determine whether the dT-mediated enhancement of base editing (B-to-A) is related to its effect on the cell cycle. To this end, we performed parallel experiments using cytarabine (Ara-C), which specifically induces S-phase arrest 27, as a control. Treatment with 100 nM Ara-C produced cell cycle arrest effects comparable to those of 5 mM dT treatment (Figure 4A). However, 100 nM Ara-C treatment did not obviously affect B-to-A editing efficiency across all editors. In the CGBE system, the C-to-A editing efficiency upon Ara-C treatment was similar to that in the PBS control (0.8% with Ara-C versus 0.9% with PBS, *p* > 0.05). By contrast, 5 mM dT increased the average C-to-A efficiency 6.3-fold (Figure 4B). Similarly, Ara-C did not affect G-to-A and T-to-A editing efficiencies in gGBE and DAF-TBE, whereas dT significantly increased G-to-A ~ 4-fold (Figure 4C) and T-to-A ~ 2-fold, respectively (Figure 4D). In terms of editing product purity, Ara-C did not obviously affect B-to-A product purity as compared to the PBS control. By contrast, dT increased the A product purity in CGBE ~ 7.2-fold (Figure 4E), in gGBE ~ 4-fold (Figure 4F), and in DAF-TBE ~ 2.2-fold (Figure 4G). Importantly, neither dT nor Ara-C caused significant cytotoxicity at the concentrations used here (Figure S8). Therefore, these results collectively demonstrated that the enhancement by dT treatment on B-to-A editing did not result from its effect on cell cycle blockage or cell viability.

### The improvement of B(C/G/T)-to-A editing by dT depends on TK1-mediated dTTP metabolism

**Figure 5:**
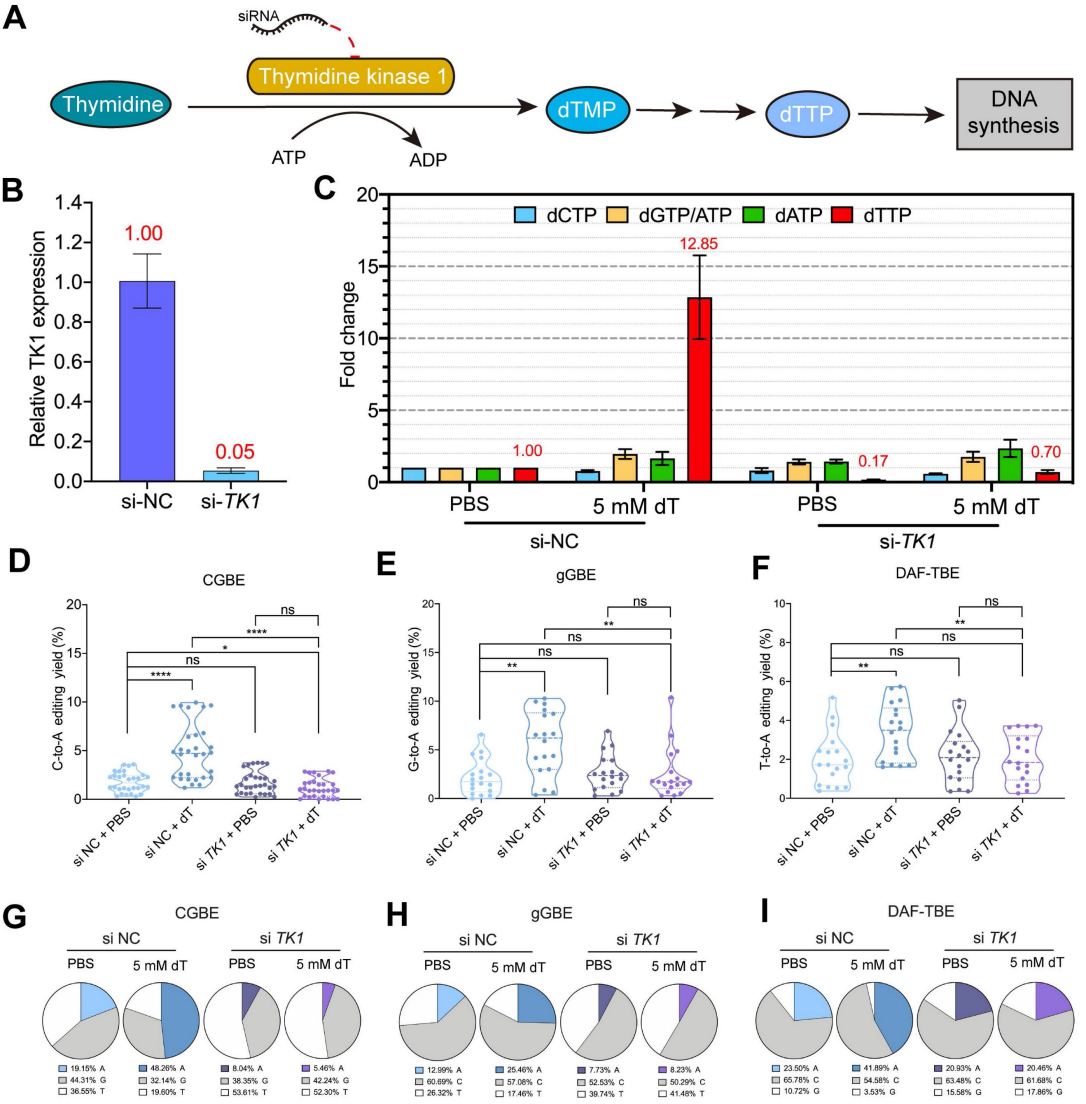
** dT metabolism regulates glycosylase base editor activity through TK1-dependent dTTP production. A.** Schematic of dT metabolic pathway: Exogenous dT is phosphorylated by cytoplasmic thymidine kinase (TK1) to dTMP, then converted to dTTP for DNA synthesis. **B.** qPCR analysis of *TK1* mRNA levels following siRNA-mediated knockdown (si-*TK1*) compared to non-targeting control (si-NC). Data are presented as mean ± s.d. (n = 3 biological replicates). **C.** Intracellular dNTP concentrations measured in si-*TK1*-treated versus si-NC-treated cells. Values represent mean ± s.d. (n = 3). Fold-change in dNTP concentrations relative to si-NC + PBS-treated control cells. Numerical values (red) indicate the magnitude of change compared to the si-NC + PBS baseline. Baseline dNTP concentrations in si-NC + PBS-treated cells were 8.14 pmol dCTP, 22.15 pmol dTTP, 7.63 pmol dATP, and 3040.82 pmol dGTP/ATP per 10^6^ cells. **D.** C-to-A editing efficiency mediated by CGBE under *TK1* knockdown (si-*TK1*) versus non-targeting control (si-NC), summarizing data from all targeted cytosine sites across five genomic loci with each point representing an independent biological replicate (**** *p* < 0.0001, ns = not significant). **E.** G-to-A editing efficiency mediated by gGBE under *TK1* knockdown (si-*TK1*) versus non-targeting control (si-NC) (** *p* < 0.01, **** *p* < 0.0001). **F.** T-to-A editing efficiency mediated by DAF-TBE under *TK1* knockdown (si-*TK1*) versus non-targeting control (si-NC) (* *p* < 0.05). **G.** Composition of CGBE editing outcomes between si-*TK1* and si-NC treated cells aggregated from five genomic loci. **H.** Composition of gGBE editing outcomes between si-*TK1* and si-NC treated cells aggregated from three genomic loci. **I.** Composition of DAF-TBE editing outcomes between si-*TK1* and si-NC treated cells aggregated from three genomic loci.

The above results of Ara-C supported our hypothesis that supplementation with dT elevates intracellular dTTP levels, which in turn facilitates T incorporation during TLS across editor-induced AP sites, thereby biasing repair toward AP-to-A outcomes via subsequent base excision repair (BER). To test this possibility, we inhibited the conversion of dT to dTTP by depleting the expression of cytosolic thymidine kinase 1 (TK1), the rate-limiting enzyme in the salvage pathway of dTTP synthesis 28 (Figure 5A). siRNA-mediated knockdown reduced *TK1* mRNA expression by ~95% (Figure 5B), which markedly blocked dTTP accumulation upon dT treatment. LC-MS analysis revealed that dT supplementation elevated intracellular dTTP levels by 11.85-fold relative to the baseline in si-NC-treated cells (Figure 5C). By contrast, *TK1* knockdown almost completely abolished this effect, with dTTP levels reducing to ~85% of the baseline, suggesting that exogenous dT cannot be efficiently converted into dTTP in the absence of TK1 (Figure 5C).

We next examined the impact of *TK1* depletion on CGBE editing outcomes. We found that si-*TK1* treatment almost completely abolished the C-to-A editing boosted by dT supplementation. In *TK1* wild-type cells (si-NC), dT treatment significantly increased the average C-to-A editing efficiency from 1.8% (PBS) to 4.9% (Figure 5D). By contrast, in *TK1*-depleted cells, dT treatment failed to enhance C-to-A editing, with the average efficiency dropping to 1.19%, even lower than that in *TK1* wild-type cells without dT treatment (1.8%) (Figure 5D), demonstrating that TK1 activity is essential for mediating the dT-dependent enhancement. A consistent trend was observed in both gGBE and DAF-TBE systems. dT increased gGBE-mediated G-to-A and DAF-TBE-mediated T-to-A editing only in *TK1* wild-type backgrounds, and these effects were abolished upon *TK1* depletion (Figure 5E-F). Similarly, the proportion of A-product edits increased only in wild-type cells after dT treatment (Figure 5G-I). For instance, in the CGBE system, dT raised the A-product purity from 19.2% to 48.3% in control cells, but no increase was observed under *TK1* knockdown (Figure 5G). Notably, *TK1* knockdown also elevated the levels of non-A edits (C-to-G and C-to-T) (Figure S9-S11), indicating that disrupting of dTTP homeostasis alters nucleotide incorporation preferences and compromises editing specificity.

### Effects of other dNs treatments on base editing outcomes

**Figure 6:**
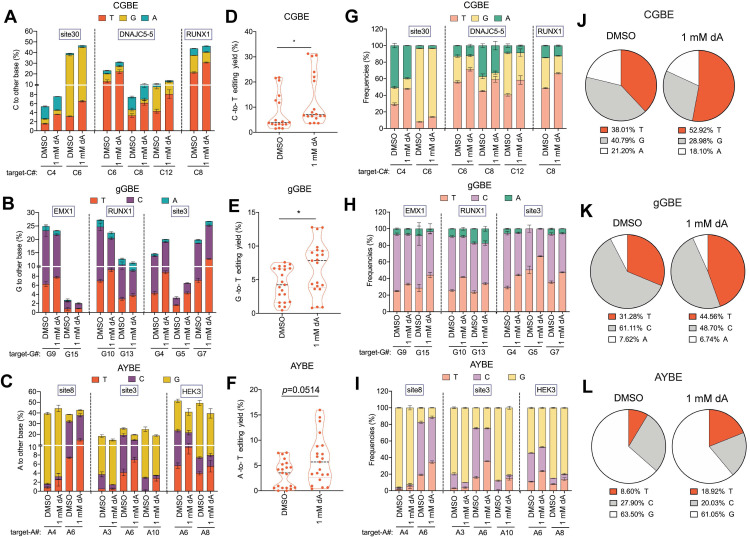
** 2'-deoxyadenosine (dA) enhances V(C/G/A)-to-T conversions in three glycosylase base editors. A.** Endogenous locus editing efficiencies of CGBE with 1mM dA in HEK293T cells. The presented data are representative of three independent experiments, and error bars represent the standard deviation of the mean. **B.** Editing efficiency of gGBE. **C.** Editing efficiency of AYBE. **D.** C-to-T editing efficiency mediated by CGBE, summarizing data from all targeted cytosine sites across three genomic loci with each point representing an independent biological replicate. **E.** G-to-T editing efficiency mediated by gGBE (* *p* < 0.05). **F.** A-to-T editing efficiency mediated by AYBE (* *p* < 0.05). **G.** Product distribution of CGBE-mediated editing. The presented data are representative of three independent experiments, and error bars represent the standard deviation of the mean. **H.** Product distribution of gGBE-mediated editing. **I.** Product distribution of AYBE-mediated editing. **J.** Composition of C-to-A, C-to-G, and C-to-T products from CGBE editing across three genomic loci. **K.** Composition of editing products from gGBE editing aggregated from three genomic loci. **L.** Composition of editing products from AYBE editing aggregated from three genomic loci.

To evaluate the generality of dNTP pool modulation on glycosylase-based base editing, we tested supplementation with dA, dC, and dG. High concentrations (5 mM) of dA and dG severely impaired cell viability, whereas dC had no detectable effect (Figure S12); therefore, subsequent experiments were conducted with 1 mM dA, 1 mM dG, or 5 mM dC. Notably, cell cycle analysis revealed that among these nucleosides, only 1 mM dG supplementation led to G1 phase accumulation, while 1 mM dA and 5 mM dC showed no detectable cell cycle effects (Figure S13). Across multiple endogenous loci and all glycosylase editors, dA treatment markedly increased both T editing efficiency and product purity. On average, C-to-T, G-to-T, and A-to-T editing efficiencies increased by 60%, 59%, and 89%, respectively (Figure 6A-F), with corresponding purities elevated by 39%, 42%, and 120% (Figure 6G-L). These findings are consistent with the notion that elevated dATP levels bias TLS polymerases to preferentially insert dATP opposite AP sites.

To assess whether the enhancing effect of dA is conserved across different cellular contexts, we tested its impact on V(C/G/A)-to-T editing mediated by the three base editors in HeLa cells. Consistent with observations in HEK293T cells, dA supplementation significantly increased G-to-T and A-to-T editing efficiency and product purity in HeLa cells, whereas the enhancement of C-to-T editing was relatively modest (Figure S14). Collectively, these findings further supported that dA can promote T product formation by modulating the intracellular dNTP pool.

However, dC supplementation did not significantly enhance G editing efficiency or purity (Figure S15), likely reflecting the high insertion specificity of cytosine, such as by REV1 polymerase, during TLS. Given that 5 mM dC showed no detectable cytotoxicity, we further tested higher concentrations (8, 10, and 20 mM) to evaluate their effects on editing outcomes. Increasing dC concentration did not lead to further improvement in C-to-G, T-to-G, or A-to-G editing in most conditions. The only exception was observed in the DAF-TBE system, where 8 mM dC modestly increased T-to-G editing purity, but this effect did not continue to rise at higher concentrations (Figure S16). These findings suggest that dC has a relatively limited impact on glycosylase-based base editing outcomes. dG supplementation did not increase AP-to-C editing efficiency but modestly improved product purity, with C proportions rising by 27%, 31%, and 19% in G-to-C, T-to-C, and A-to-C edits, respectively (Figure S17). Together, these results indicate that exogenous nucleosides differentially modulate base editing outcomes by altering intracellular dNTP balance, with dT and dA showing the most pronounced enhancement.

### Improvement of modeling disease related C-to-A mutations by dT treatment

**Figure 7:**
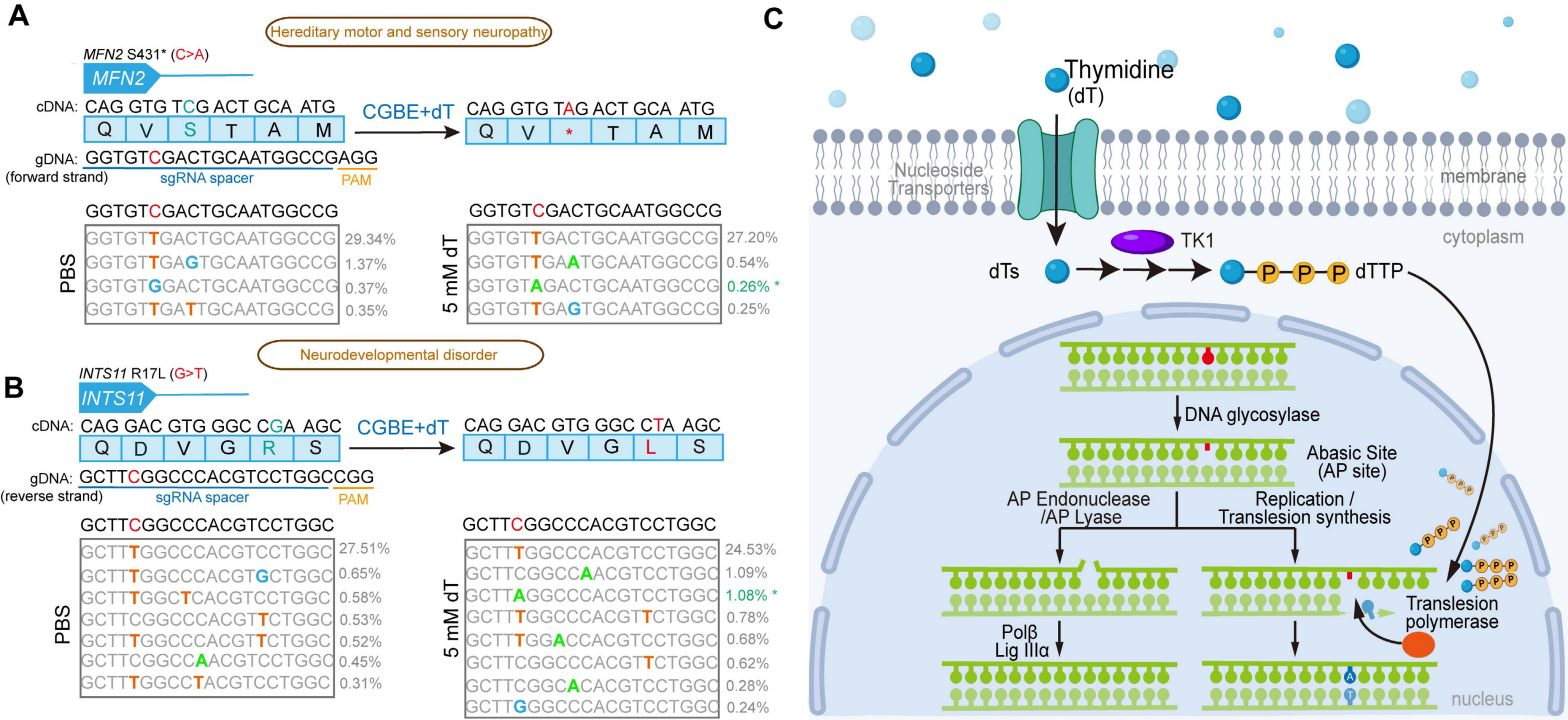
** Construction of C-to-A pathogenic SNVs in HEK293T using CGBE. A.** Pathogenic single nucleotide variation (SNV) engineering at the *MFN2* locus. **B.** Pathogenic SNV generation at the *INTS11* locus. The blue box displays the amino acid sequence with corresponding cDNA sequence above. Below the blue box is the sgRNA-targeted genomic sequence with the target C highlighted in red. The sequence underlined in blue indicates the sgRNA spacer, and the sequence underlined in yellow denotes the PAM motif (NGG) for SpCas9. Above the gray box is the original genomic sequence (target C in red), while inside the gray box are high-throughput sequencing results showing various edited sequences, with successful C-to-A conversions marked by asterisks on the right. The data represent the average of three independent biological experiments. **C.** Molecular mechanism of dT-enhanced B-to-A editing by glycosylase base editors. Exogenous thymidine (dT) enters cells through nucleoside transporters and is sequentially phosphorylated to dTTP by cytoplasmic thymidine kinase (TK1). At AP sites generated by glycosylase editors, elevated dTTP levels promote B-to-A editing through three key steps: First, translesion synthesis (TLS) DNA polymerases are recruited to AP sites and preferentially incorporate dTTP due to its increased cellular concentration. Second, the incorporated dTTP forms an AP site •T mismatch with the template strand. Third, cellular repair systems convert the AP site •T mismatch into an A•T base pair, ultimately achieving efficient AP site-to-A editing.

To evaluate the potential of dT to enhance targeted glycosylase base editing for the generation of pathogenic single-nucleotide variants (SNVs), we selected two C-to-A disease-associated SNV sites for testing: *MFN2*, associated with hereditary motor and sensory neuropathy, and *INTS11*, associated with neurodevelopmental disorders. At the *MFN2* locus (c.1292C > A), no detectable C-to-A editing was observed in the control group receiving only the CGBE, whereas dT treatment achieved an editing efficiency of 0.26% (Figure 7A). Similarly, at the *INTS11* locus (targeting the C-to-A change complementary to c.50G > T), PBS-treated cells also showed no C-to-A editing, whereas dT supplementation enabled 1.08% editing (Figure 7B). These results demonstrate that dT treatment can overcome the inherent limitations of existing base editors, successfully inducing C-to-A mutations at genomic sites that were previously inaccessible, highlighting its unique utility for biomedical research and potential therapeutic applications.

To assess the potential impact of exogenous dT supplementation on genomic stability, we performed deep mutational analysis of the edited endogenous target sites shown in Figures 2 and 3, together with their flanking regions. These regions are expected to be most directly influenced by alterations in intracellular dNTP pools and the activity of DNA polymerases, including TLS polymerases, and thus serve as sensitive indicators of broader mutational spectrum. We observed no significant difference in mutation frequency between dT-treated and PBS control groups across these regions (Figure S18-S20), indicating that, under the conditions tested, dT supplementation did not lead to a detectable increase in genome-wide mutational burden.

In summary, our findings demonstrate that intracellular dNTP levels can be modulated to influence the product outcomes of glycosylase-based base editors, with dT being the most robust (Figure 7C). Exogenous dT enters cells via nucleoside transporters and is phosphorylated by TK1 to form dTMP, which is then converted to dTTP. At AP sites generated by glycosylase editors, elevated dTTP levels promote B-to-A editing via the following mechanism: (1) TLS DNA polymerases are recruited to the AP site and preferentially incorporate dTTP due to its higher intracellular concentration; (2) the incorporated dTTP in turn forms an AP site •T mismatch with the template strand; (3) the cellular repair machinery subsequently converts the AP site •T mismatch into an A•T base pair possibly via base excision repair pathway, resulting in AP-to-A editing.

## Discussion

In summary, we discovered a novel strategy for improving glycosylase base editing by modulating nucleotide metabolism. We found that supplying exogenous nucleosides can elevate the intracellular levels of the corresponding nucleotides, thereby shifting the product distribution of glycosylase-based base editors to improve both the efficiency and purity of the desired base conversion. This effect was most pronounced with thymidine, as thymidine treatment markedly enhanced B(C/G/T)-to-A directional editing efficiency mediated by multiple editors, including CGBE, gGBE, and DAF-TBE, and was consistently observed in both HEK293T and HeLa cells. We then demonstrated that the enhancement depended on the salvage pathway in which TK1 phosphorylates dT to generate dTTP but was independent of cell-cycle arrest. Therefore, our work provided a new strategy for optimizing base editing, extending interventions beyond conventional protein engineering to the regulation of the intracellular metabolic pathways. Compared with direct engineering of glycosylases, Cas protein domains or TLS polymerases, dT-mediated metabolic intervention required no redesign of the editor components, offering a convenient “plug-and-play” approach for the improvement of existing base editors.

Previous studies indicated that glycosylase-based base editors rely on TLS polymerases to insert nucleotides opposite to AP sites, generating diverse base substitution outcomes that are determined by the intrinsic substrate preferences of different TLS polymerases 6, 12, 16. Replicative polymerases such as Pol δ and Pol ε are relatively high-fide because of highly constrained and sequence-specific active sites, rendering them poorly tolerant of non-instructional lesions such as AP sites. Consequently, when replicative polymerases encounter an AP site during DNA synthesis, replication is typically stalled 29-31. In contrast, TLS polymerases exhibit relaxed substrate specificity, enabling them to bypass DNA lesions, including non-templating AP sites. In the mammalian genomes, Y family DNA polymerases were the most extensively characterized TLS polymerases. And many members of this family were capable of bypassing AP site and possessing different substrate specificities 23. Structural and biochemical analyses revealed that Pol η preferred inserting dATP or dTTP during AP site bypass 23, 32, Pol κ preferred dCTP and dATP 23, Pol ι prefers dGTP and dTTP 24, and REV1, functioning as a specialized deoxycytidine transferase, specifically catalyzed dCTP insertion 20.

Consistent with this notion, gain- or loss-of-function of individual TLS polymerases significantly altered editing outcomes during glycosylase-based base editing. Overexpression of Pol η in mammalian cells shifted AYBE editing toward A-to-T conversion, supporting its role in inserting A/T opposite AP sites 6, whereas Rev1 deletion in yeast abolished C-to-G editing mediated by CGBE 16. These observations supported the conclusion that AP-site repair is dominated by TLS polymerases rather than replicative polymerases. In line with this model, our observation that modulation of the intracellular dNTP pool reshaped editing outcomes further suggested that the activity or nucleotide preference of TLS polymerases could be functionally tuned in cells.

The functional redundancy of TLS polymerases likely resulted in the diversity of products generated by different glycosylase editors. Although different editing outcomes were observed with each editor, the C outcome seemed to be preferred in mammalian cells, suggesting that in mammalian cells, dGTP incorporation is intrinsically efficient 6-11. Consistent with this notion, in our observations, AP to C outcomes were relatively insensitive to external metabolic perturbations compared with other outcomes, as revealed by dT and dG treatment. By contrast, the efficiency of AP-to-T editing was highly sensitive to intracellular dTTP levels. Thymidine supplementation markedly reduced both the efficiency and purity of C-to-T and G-to-T editing, whereas TK1 knockdown had the opposite effect. This observation suggested that the polymerases responsible for inserting A or T opposite AP sites might belong to the same class or operate in a coupled manner. Elevated dTTP levels could bias their activity toward T incorporation, thereby decreasing AP-to-T outcomes while enhancing AP-to-A editing products. Interestingly, reanalysis of high-throughput sequencing data from a previous investigation on the effect of DNA repair pathway on CGBE editing revealed that knockdown of Pol η, Pol ι, or Pol θ slightly reduced the proportion of A outcomes in CGBE (data not shown) 33. These polymerases, especially Pol η, are capable of bypassing AP site by inserting either A or T. Therefore, we hypothesized that these three TLS polymerases might contribute to dTTP insertion opposite AP sites, thereby contributing to the enhancement of B-to-A outcomes by dTTP.

Notably, supplementation with dT did not significantly increase the overall base-editing efficiency and even led to a modest reduction at certain loci (Figure 2B). This might because our metabolic modulation strategy did not increase the number of initial editing events — namely, the formation of AP sites by glycosylases — but rather to reshape the repair outcomes at pre-existing AP sites. The slight decrease in overall efficiency observed in some cases might be attributed to competition between repair pathways within the cell. Besides TLS repair, BER pathway might also contribute to the AP site repair 34, 35. When the concentration of a specific dNTP (such as dTTP) increases and strongly biases a particular TLS polymerase, it may slightly suppress other TLS events, potentially increasing BER repair that restores the lesion to its original sequence, thereby reducing overall editing efficiency.

Importantly, the “metabolic modulation” strategy proposed here differs fundamentally from approaches that rely on engineering the editor proteins themselves 36-41. Instead, it directly targets cellular metabolism, making it experimentally straightforward and readily compatible with existing base editors. As such, this strategy offers a simple and rapid route to enhance editing efficiency for both disease modeling and therapeutic applications. Nevertheless, the absolute efficiency of this approach may still need to be improved in certain contexts. Future optimization could be achieved through combinatorial strategies, including the use of high-performance editor variants, fine-tuning TLS polymerase activity, or improving chromatin accessibility.

Despite its effectiveness in improving targeted editing outcomes, the potential limitations of metabolic intervention must also be carefully considered. Sustained imbalance of the dNTP pool is intrinsically mutagenic, as aberrant dNTP levels are frequently associated with mutation accumulation in cancer cells or under conditions of DNA damage 42-46. Notably, under the experimental conditions used in this study, we did not detect an obvious increase in mutations within the flanking regions of the edited sites, which are expected to be particularly sensitive to dNTP imbalance. This observation suggests that transient dT supplementation does not generally increase genome-wide mutational events. However, to advance this strategy toward clinical translation, future studies will be required to systematically evaluate global mutation burden and other safety issues associated with dT supplementation.

## Materials and Methods

### Plasmid construction

The plasmids gGBE and AYBE were kindly provided by Dr. Yang Hui (Institute of Neuroscience, Center for Excellence in Brain Science and Intelligence Technology, Chinese Academy of Sciences) 6, 7; and the plasmid DAF-TBE was kindly provided by Dr. Bi Changhao (Tianjin Institute of Industrial Biotechnology, Chinese Academy of Sciences) 9. The CGBE plasmid was constructed by modifying the BE4 backbone (Addgene, #100802). The UGI domain was excised from the BE4 plasmid via PCR amplification. The resulting linearized vector was then circularized using the ClonExpress Ultra One Step Cloning Kit V3 (Vazyme) according to the manufacturer's protocol. sgRNA plasmids were constructed through inserting oligos containing desired spacers into Bbs1 digested empty plasmids with SpCas9 scaffold. The ligated products were transformed into competent *E. coli* DH5α cells, and positive clones were screened by colony PCR followed by Sanger sequencing. Oligos used to generate sgRNAs were listed in Table S1.

### C-to-A editing reporter construction

Reporter system design and vector construction. To precisely capture C-to-A editing events, we developed a reporter system comprising an mCherry-EGFP fusion gene driven by the CMV promoter. A chromophore-deficient EGFP reporter was engineered by introducing the Y66D mutation (TCA > GCA) within the S65-Y66-G67 chromophore motif. Functional chromophore activity is restored exclusively when a corrective G-to-T edit (corresponding to C-to-A on the antisense strand) reverts GCA to the wild-type TCA sequence. The Tol2-mCherry-EGFP-IRES-PuroR vector was constructed via multi-fragment seamless cloning using the ClonExpress Ultra One Step Cloning Kit V3 (Vazyme). The Y66D point mutation was subsequently introduced directly into this vector by PCR. The linearized product was circularized using the same ClonExpress kit. The EGFP Y66N mutant control was generated using an identical procedure.

Stable cell line generation and validation. Stable HEK293T cells expressing the mCherry-EGFP(Y66D) fusion were established by co-transfecting the Tol2-mCherry-EGFP(Y66D)-IRES-PuroR plasmid with a transposase expression vector at a 1:1 molar ratio using LipoMax DNA Transfection Reagent (Sudgen). At 24 h post-transfection, the medium was replaced with complete medium containing 3 μg/mL puromycin for selection over 7-10 days until all control cells died. The resulting cell line exhibited red fluorescence (mCherry) but no detectable green fluorescence (EGFP) by fluorescence microscopy, confirming the chromophore-deficient phenotype.

### Cell culture and transfection

HEK293T cells were cultured in Dulbecco's Modified Eagle Medium (DMEM; Biopico) supplemented with 10% (v/v) fetal bovine serum (FBS; ExCell) and 1% penicillin/streptomycin (100 U/mL penicillin, 100 μg/mL streptomycin; Sperikon) at 37 °C in a humidified 5% CO2 incubator.

Cells were seeded in 96-well plates (BIOFIL) and cultured for 16-18 h until ~80% confluency prior to transfection. Cells were then co-transfected using LipoMax DNA transfection reagent (Sudgen) according to the manufacturers protocol with a mixture of plasmids including 300 ng of base editor plasmid, 100 ng of target-specific sgRNA plasmid, and 30 ng of a selection plasmid conferring resistance to either hygromycin (Meilunbio) or puromycin (Beyotime). Transfected cells were harvested 72 h post-transfection for genomic DNA extraction.

### HEK293T siRNA transfection

HEK293T cells were plated in 24-well plates (BIOFIL) at 5×10^4^ cells/well in DMEM + 10% FBS. After 16 h (~60% confluency), cells were transfected with CALNP™ RNAi reagent (D-Nano Therapeutics) and 25 pmol TK1 siRNA (si-TK1: 5′-ACAAGTGCCTGGTGATCAA-3′ 47) or non-targeting Control (NC) siRNA following the manufacturer's instructions. 24 h after transfection, the medium was replaced with fresh DMEM + 10% FBS. 48 h after transfection, cells were washed with PBS and resuspended using 1 × Trypsin 0.25%-EDTA solution (Sperikon), and then replated in 96-well plates at 2 × 10^4^ cells/well in DMEM + 10% FBS. At 16-24 h post-seeding (~60-80% confluency), cells were transfected with 0.7 μL LipoMax DNA transfection reagent (Sudgen) and 300 ng of base editor plasmid, and 100 ng of sgRNA plasmid. 6 h after plasmid transfection, cells were retransfected with CALNP™ RNAi reagent and 5 pmol of the same siRNAs used initially.

### Nucleoside analog treatment in HEK293T cells

Drug stock solutions were prepared as follows: thymidine (dT; Beyotime) and 2'-deoxycytidine (dC; OriLeaf) were dissolved in PBS to 100 mM; 2'-deoxyadenosine (dA; OriLeaf) and 2'-deoxyguanosine (dG; OriLeaf) were prepared in DMSO at 1 M and 500 mM, respectively; and cytarabine (Ara-C) was dissolved in DMSO to 50 mM. Working concentrations were achieved by direct dilution in culture medium immediately before use. For Ara-C, stepwise dilution was performed to attain lower concentrations.

For Figure 1, Figure 2, Figure 3, Figure S4 and Figure S5: At 6 h post-transfection, the medium was replaced with dT-containing medium at specified concentrations and maintained until genomic DNA extraction. Controls received equivalent volumes of PBS in medium.

For Figure 4: At 6 h post-transfection, cells were treated with medium containing 5 mM dT or 100 nM Ara-C until harvest. Controls received medium with DMSO volumes equivalent to that in Ara-C treatment.

For Figure 5: At 24 h post-second siRNA transfection, medium containing 5 mM dT was administered until harvest. Controls received medium with equivalent PBS volumes.

For Figure 6 and Figure S14-S17: At 6 h post-transfection, the medium was replaced with dA, dC, or dG-containing medium at specified concentrations and maintained until genomic DNA extraction. Controls received equivalent volumes of PBS or DMSO in medium.

### Genomic DNA extraction

Cells were cultured for 72 h after transfection before genomic DNA was isolated. Cells were washed once with PBS and lysed with gDNA lysis buffer: 10 mM Tris-HCl, pH 8.0; 0.1 M NaCl; 0.01 mM EDTA; 100 μg/mL proteinase K (New England BioLabs) at 55 °C for 45 min, followed by enzyme inactivation at 95 °C for 15 min.

### Flow cytometry analysis

Seventy-two hours after transfection, expression of mCherry and EGFP fluorescence was analyzed by BD FACSAria™ III. Flow cytometry results were analyzed with NovoExpress. The gating strategy in the identification of mCherry-positive and EGFP-positive cells for on-target editing efficiency evaluation is supplied in Figure S2.

### Cell cycle assay

HEK293T cells were plated in 6-well plates (BIOFIL) at 8×10^5^ cells/well in DMEM + 10% FBS. After 16 h (~80% confluency), cells were treated with either dT (dissolved in PBS at 5 mM) or Ara-C (dissolved in DMSO at 100 nM) for 24 h. Cells were then harvested and fixed in ice-cold 70% ethanol overnight at -20 °C. After fixation, cells were pelleted by centrifugation at 1000 × *g* for 5 min, washed twice with 2 mL cold PBS, and repelleted under identical conditions. Next, cells were incubated with 100 μL RNase A (final concentration 200 μg/mL) for 30 min at 37 °C, washed once with PBS, and centrifuged as above. Cells were resuspended in 500 μL PBS and stained with 5 μL 7-AAD (BestBio) for 30-60 min at 4 °C. DNA content was analyzed using a flow cytometer (excitation: 488 nm; emission: 647 nm), and cell cycle distribution was analyzed with Flowjo software.

### CCK-8 cell viability assay

Cell viability was assessed using a Cell Counting Kit-8 (CCK-8) assay (Oriscience Biothechnology). Briefly, HEK293T cells were seeded in 96-well plates at a density of 20000 cells per well in 100 µL of complete medium and allowed to adhere overnight in a 37 °C, 5% CO_2_ incubator. The following day, the culture medium was replaced with 100 µL of fresh medium containing the test compounds at various concentrations; for compounds dissolved in DMSO, the final DMSO concentration was normalized to less than 0.1% across all wells, including the negative control. After 24 h of treatment, 10 µL of CCK-8 reagent was added to each well, followed by incubation for 1 h at 37 °C. Absorbance was measured at 450 nm using a microplate reader (BioTek EPOCH2). Cell viability was calculated using the following formula: [(As - Ab) / (Ac - Ab)] × 100%, whereas represents the absorbance of the test compound well, Ac represents the absorbance of the DMSO-treated control well, and Ab represents the absorbance of the blank control well (medium only, without cells).

### qPCR analysis

Total RNA was isolated using the Cell Total RNA Isolation Kit (FOREGENE) according to the manufacturer's instructions. RNA concentration and purity were determined using a microvolume spectrophotometer (ALLSHENG Nano-200D), with A260/A280 ratios of 2.0-2.2 considered acceptable. Reverse transcription was performed with 1 μg of total RNA using the Evo M-MLV RT Mix Kit (Accurate Biotechnology) under the following conditions: 42 °C for 15 min, 85 °C for 5 s.

Quantitative PCR was carried out in triplicate using SYBR Green Premix Pro Taq HS qPCR Kit (Accurate Biotechnology) on a SLAN-96S real-time PCR system (Shanghai Hongshi Medical Technology). Each 20 μL reaction contained: 10 μL SYBR Green Mix, 0.8 μL each primer (10 μM), 2 μL cDNA template, and 6.4 μL nuclease-free water. The primer sequences were:

*TK1* (Forward: 5′-GCCAAAGACACTCGCTACAG-3′, Reverse: 5′-CCCCTCGTCGATGCCTATG-3′).

*ACTB* (β-actin) (Forward: 5′-CCTGGCACCCAGCACAAT-3′, Reverse: 5′-GGGCCGGACTCGTCATAC-3′).

Cycling parameters:

95 °C for 30 s; 40 cycles of 95 °C for 5 s, 60 °C for 30 s; followed by a melt curve analysis (65 °C to 95 °C, increment 0.5 °C / 5 s). Gene expression was normalized to ACTB and calculated using the 2^-ΔΔCt^ method.

### Extraction and measurement of cellular dNTPs

Deoxynucleoside triphosphate (dNTP) standards (dATP, dTTP, dCTP, dGTP; Beyotime Biotechnology) were serially diluted in 70% methanol from 100 mM stocks to concentrations of 0.1-30 μM and stored at -80 °C ≤ 2 weeks. For biological samples, HEK293T cells (1 × 10^6^ cells/well in 6-well plates) were trypsinized and resuspended in 1 mL PBS. A 20 μL aliquot was mixed with trypan blue for viable cell counting using a hemocytometer. The remaining suspension was centrifuged (1,000 × *g*, 5 min, 4 °C). After supernatant removal, pellets were lysed in 200 μL 70% methanol with vortexing (30 s) and ice incubation (15 min). Cleared lysates (12,000 × *g*, 10 min, 4 °C) were analyzed immediately or stored at -80 °C.

dNTPs were analyzed by liquid chromatography-tandem mass spectrometry (LC-MS/MS). LC-MS/MS was performed using a Qtrap 5500 triple quadrupole mass spectrometer (AB Sciex, USA) coupled with an ultra-fast liquid chromatography (UFLC) system (Shimadzu, Japan) comprising a SIL-30AC autosampler, LC-30AD binary pump, CBM-20A system controller, and CTO-20AC column oven. Separation was achieved on a Waters Acquity UPLC® BEH C18 column (1.7 µm, 2.1 × 50 mm) maintained at 40 °C with the autosampler temperature set at 15 °C; the mobile phase consisted of 0.01% ammonium hydroxide in water (A) and acetonitrile (B) delivered at a flow rate of 0.7 mL·min^-1^ under the following gradient program: 10% B at initial conditions, increased to 90% B by 0.7 min, held until 1.7 min, with a total run time of 2.0 min per injection, an equilibration time of 0.6 min, and an injection volume of 0.5 µL. Detection was performed using electrospray ionization in negative ion mode with multiple reaction monitoring (MRM); the ion source parameters were set as follows: capillary voltage -4.5 kV, source temperature 500 °C, and declustering potential 100 V, with specific MRM transitions, dwell times (0.1 s per transition), and collision energies optimized for each dNTP as follows: dCTP 466.1→159.1 (CE -32 eV), dTTP 481.1→159.1 (CE -33 eV), dATP 490.1→159.1 (CE -36 eV), and dGTP 506.1→159.1 (CE -33 eV). The signal at m/z 506.1→159.1 represents a composite of both dGTP and ATP, as they are chromatographically co-eluted and spectrometrically indistinguishable due to their identical mass.

### High-throughput DNA Sequencing and data analysis

Genomic DNA was extracted 72 h after transfection. Cells for Figure 1D and Figure S4D were treated with hygromycin starting at 30 h post-transfection until harvest, while all other sequencing experiments used puromycin selection throughout. The extracted DNA was amplified by PCR using 2 × Phanta UniFi Master Mix (Vazyme). The PCR reaction included 2 μL of cell lysate, and 0.4 μM of forward and reverse primers in a final reaction volume of 50 μL. Genomic regions of interest were amplified by PCR with primers flanked with different barcodes. PCR reactions were performed as follows: 98 °C for 30 s, then 35 cycles of (98 °C for 10 s, 60 °C for 10 s, and 72 °C for 8 s), followed by a final 72 °C extension for 5 min. The PCR products were purified with GeneJET Gel Extraction Kit (Thermo scientific) and quantified with microvolume spectrophotometer (ALLSHENG Nano-200D). Samples were subjected to Illumina sequencing (PE150) by Sangon Biotech (Shanghai) Co., Ltd., China. Editing efficiency was quantified with CRISPResso2 48 and the threshold of editing activity was set to above 0.2%.

### Statistical analysis

Data are presented as mean ± standard deviation (SD) of at least three independent biological replicates. Statistical analyses were performed using GraphPad Prism 8. For comparisons between two groups under normal distribution, two-tailed Student's t-test was applied. For multi-loci editing data, the Mann-Whitney U test was used. Statistical significance was defined as *p* < 0.05, with the following annotation * *p* < 0.05, ** *p* < 0.01, *** *p* < 0.001, **** *p* < 0.0001.

## Supplementary Material

Supplementary figures and tables.

## Abbreviations

DSB: DNA double-strand break
TLS: translesion synthesis
AP site: apurinic/apyrimidinic site
SNV: single nucleotide variation
UGI: uracil-DNA glycosylase inhibitor
BER: base excision repair
TK1: thymidine kinase 1
dT: thymidine
dA: 2'-deoxyadenosine
dG: 2'-deoxyguanosine
dC: 2'-deoxycytidine
Ara-C: cytarabine
CGBE: C•G-to-G•C base editor
gGBE: glycosylase-based guanine base editor
DAF-TBE: deaminase-free base editors for thymine
AYBE: adenine transversion base editor
REV1: REV1 DNA-directed polymerase
Pol κ: DNA polymerase kappa
Pol ι: DNA polymerase iota
Pol η: DNA polymerase eta
Pol θ: DNA polymerase theta
dNTP: deoxynucleoside triphosphate
dTTP: deoxythymidine triphosphate
dATP: deoxyadenosine triphosphate
dCTP: deoxycytidine triphosphate
dGTP: deoxyguanosine triphosphate
SpCas9: *Streptococcus pyogenes* Cas9
sgRNA: single-guide RNA
IRES: internal ribosome entry site
LC-MS/MS: liquid chromatography-tandem mass spectrometry
HTS: high throughput sequencing
