# Supplementary material for: Harnessing nucleotide metabolism to control glycosylase base editing outcomes
Source: Theranostics. 2026 Feb 4;16(8):4411–26. doi: 10.7150/thno.125705 (PMC12906143; doi:10.7150/thno.125705)
Supplement: Supplementary file 1 — Supplementary figures and tables. [file thnov16p4411s1.pdf]

Supplementary Materials for

Harnessing Nucleotide Metabolism to Control Glycosylase Base Editing Outcomes

Supplementary Text

Figure S1 to S20

Tables S1

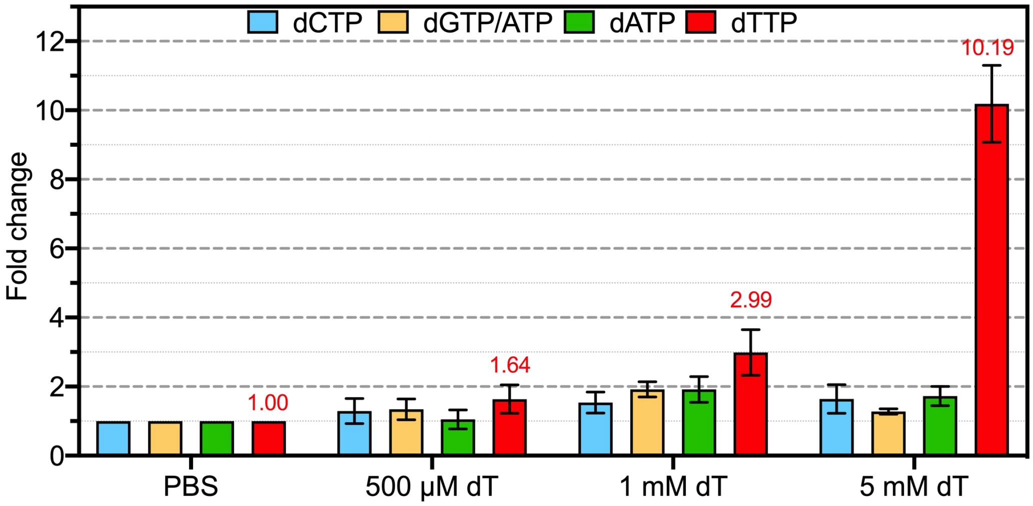

Figure. S1. dT-dependent modulation of dNTP pool concentrations in HEK293T cells. Quantification of intracellular dNTP levels following treatment with PBS (control) or increasing dT concentrations (500 μM to 5 mM). Bar graphs depict fold-change in dNTP concentrations normalized to PBS-treated cells, with numerical values (red) indicating the magnitude of increase. Baseline dNTP concentrations in PBS-treated cells were 25.28 pmol dCTP, 62.99 pmol dTTP, 25.44 pmol dATP, and 9535.06 pmol dGTP/ATP (dGTP overlapped with ATP and they were analyzed as a composite) per 10^6 cells. Error bars represent standard deviation from three independent biological replicates.

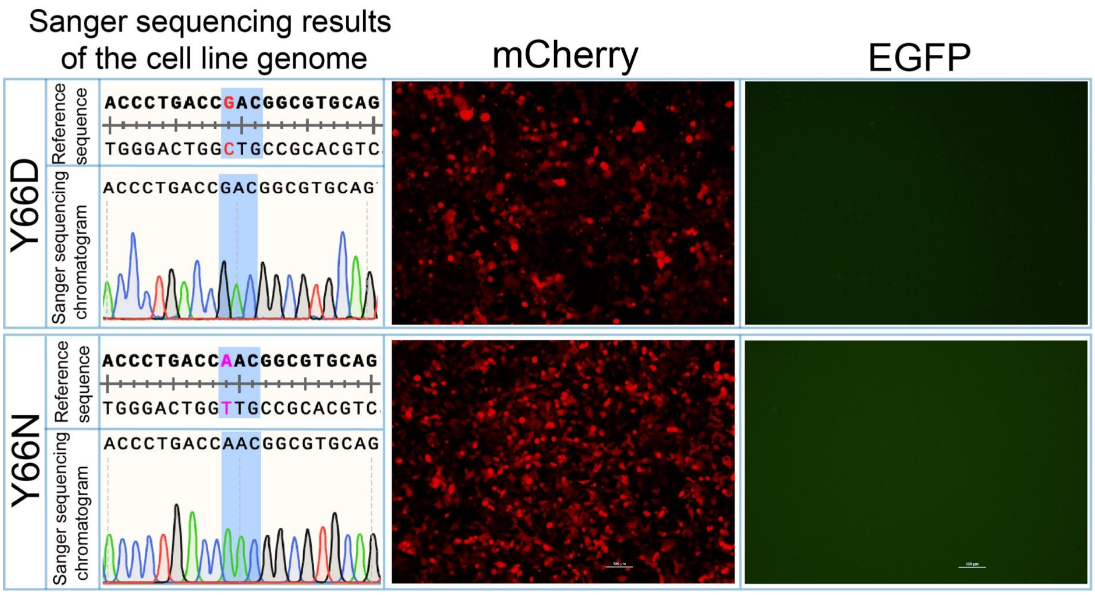

Figure. S2. Characterization of EGFP Y66D and Y66N mutant cell lines. Left panel: Sanger sequencing chromatograms of genomic DNA from HEK293T cells engineered to carry EGFP Y66D (top, target C-to-G mutation marked in red) or Y66N (bottom, target C-to-A mutation marked in pink) mutations. Right panel: Fluorescence microscopy images demonstrating loss of green fluorescence in both Y66D and Y66N mutant cell lines. Scale bar: 100 μm.

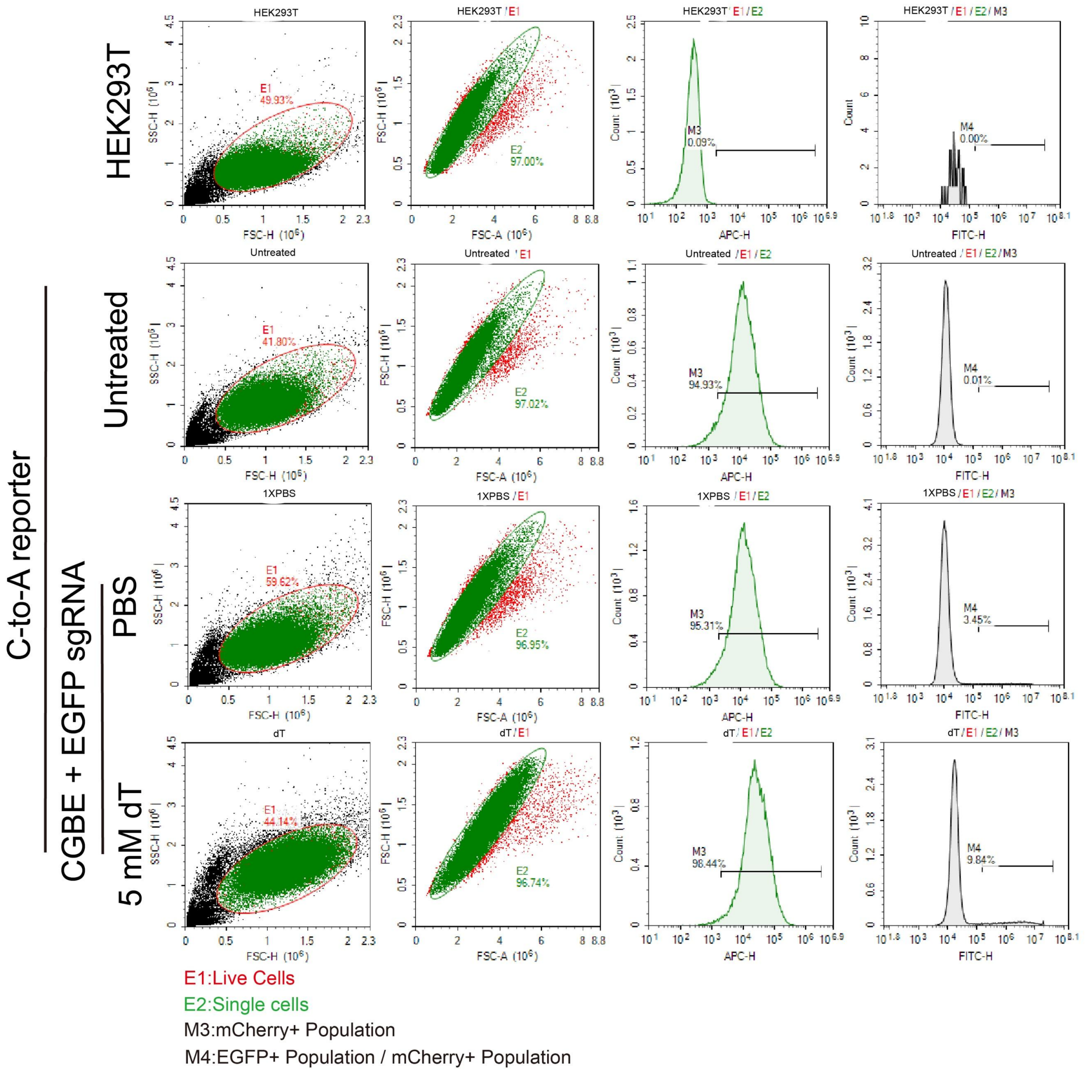

Figure. S3. Flow cytometry analysis of C-to-A editing efficiency in reporter cells. Flow cytometry was performed to assess C-to-A editing efficiency by first gating live, single cells based on FSC/SSC profiles and FSC-A vs FSC-H characteristics, followed by identification of reporter cells using mCherry fluorescence (APC-H channel) and edited cells via induced EGFP signal (FITC-H channel), with thresholds set relative to HEK293T and untreated reporter cells. Editing efficiency was calculated as the percentage of EGFP-positive cells within the mCherry-positive population.

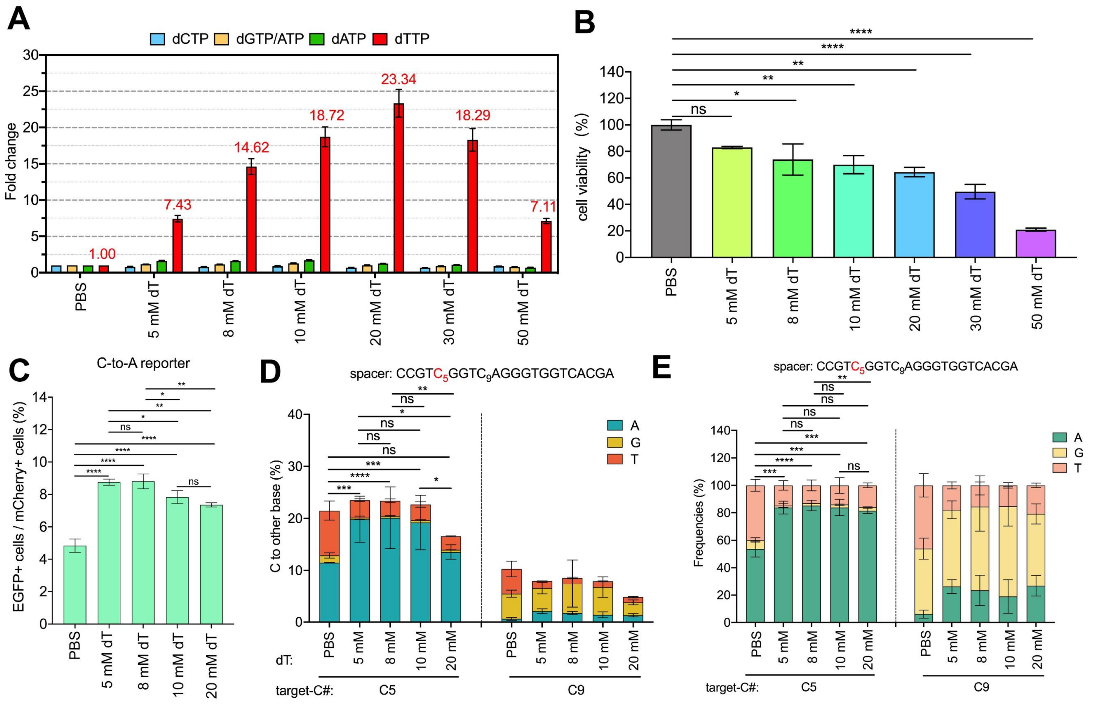

Figure. S4. Effects of a dT concentration gradient on intracellular dNTP pools, cell viability, and genome editing outcomes. A. Intracellular dNTP levels after PBS or dT treatment (5-50 mM). Bars show fold-change relative to PBS-treated cells with fold increase in red. Baseline dNTPs (per 10^6 PBS-treated cells) were 48.22 pmol dCTP, 255.95 pmol dTTP, 105.05 pmol dATP, and 42952.64 pmol dGTP/ATP. Error bars show standard deviation from three replicates. **B.** Cell viability was measured by CCK-8 assay after 24 h treatment with PBS or dT (5-50 mM). Data are mean ± s.d. percentage relative to the PBS control (100%) from three independent experiments. **C.** Percentage of EGFP-positive cells after treatment with PBS or a gradient of dT concentrations. Data are mean ± s.d. (n = 3). (* $p < 0.05$, ** $p < 0.01$, **** $p < 0.0001$, ns = not significant). **D.** C-to-D (A/G/T) conversion frequencies at EGFP Y66D locus quantified by deep sequencing. Data are presented as mean ± s.d. (n = 3). * $p < 0.05$, ** $p < 0.01$, *** $p < 0.001$, **** $p < 0.0001$, ns = not significant. **E.** Editing product distributions comparing PBS with dT. The presented data are representative of three independent experiments, and error bars represent the standard deviation of the mean (** $p < 0.01$, *** $p < 0.001$, **** $p < 0.0001$, ns = not significant).

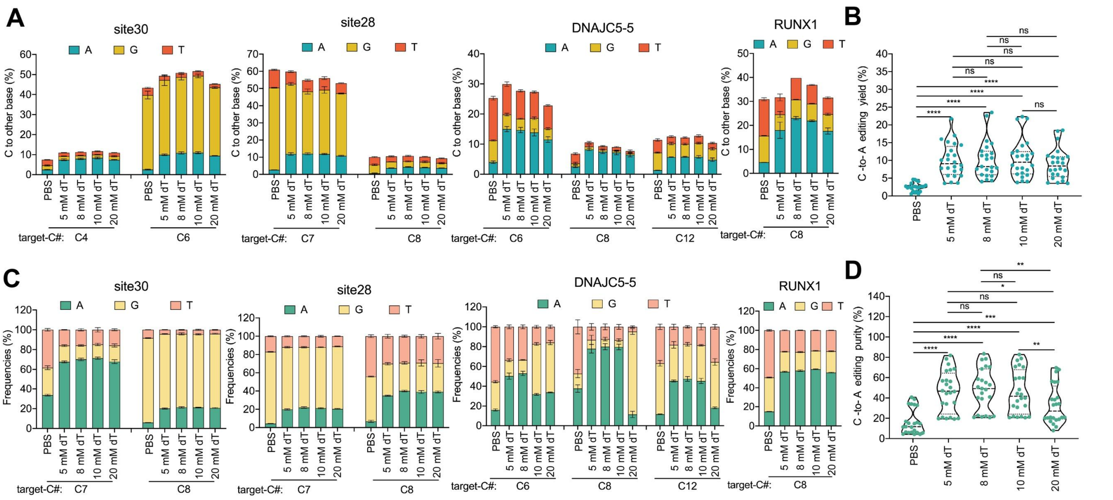

Figure. S5. Effects of graded dT concentrations on the efficiency and purity of CGBE-mediated editing at endogenous loci in HEK293T. A. Endogenous locus editing efficiencies of CGBE with graded dT concentrations in HEK293T cells. Data represent mean ± s.d. of three independent experiments. **B.** The C-to-A editing efficiencies across all replicates and target sites. Data for each editing outcome are pooled from all four endogenous loci and three biological replicates (**** $p < 0.0001$, ns = not significant). **C.** Product distribution of CGBE-mediated editing with different dT concentrations. Data are shown as mean ± s.d. from three independent experiments. **D.** The C-to-A editing purity across all replicates and target sites (* $p < 0.05$, ** $p < 0.01$, *** $p < 0.001$, **** $p < 0.0001$, ns = not significant).

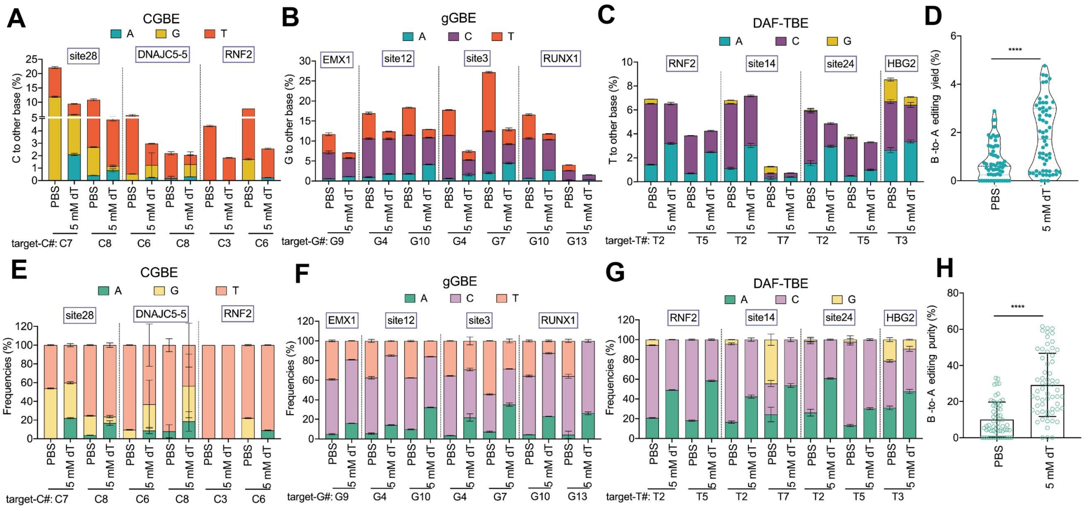

Figure. S6. Effects of 5mM dT on B-to-A editing outcomes at endogenous loci in HeLa cells. A. Endogenous locus editing efficiency following treatment with PBS or 5mM dT. Data represent mean ± s.d. of three independent experiments. **B.** B-to-A editing efficiencies across all replicates and target sites. Data for each editing outcome are pooled from all eleven endogenous loci and three biological replicates (**** $p <$ 0.0001, ns = not significant). **C.** Endogenous locus product distribution after treatment with PBS or 5mM dT. Data are shown as mean ± s.d. from three independent experiments. **D.** B-to-A editing purity across all replicates and target sites (**** $p <$ 0.0001, ns = not significant).

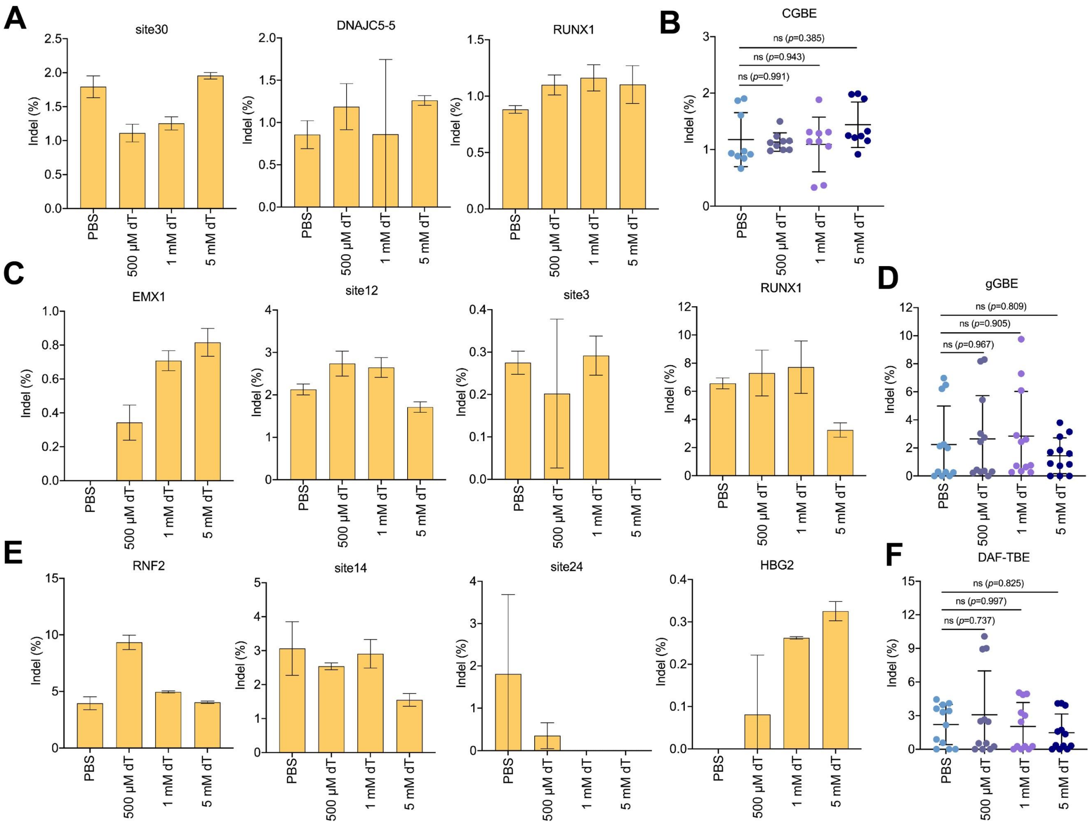

Figure. S7. Effects of dT on indel frequencies generated by glycosylase base editors. A. Indel frequencies induced by CGBE at three genomic loci, comparing dT-treated versus PBS control groups (mean ± s.d., n = 3 biological replicates). **B.** Scatter plot showing individual replicate data points from panel A, with each point representing one biological experiment (ns = not significant). **C.** Indel frequencies produced by gGBE at four target sites under dT versus PBS treatment. **D.** Scatter plot of replicate-level gGBE data from panel C, with each point corresponding to an independent experiment (ns = not significant). **E.** DAF-TBE-generated indel frequencies at four genomic loci in dT versus PBS conditions). **F.** Scatter plot displaying experimental replicates of DAF-TBE data shown in panel E (ns = not significant).

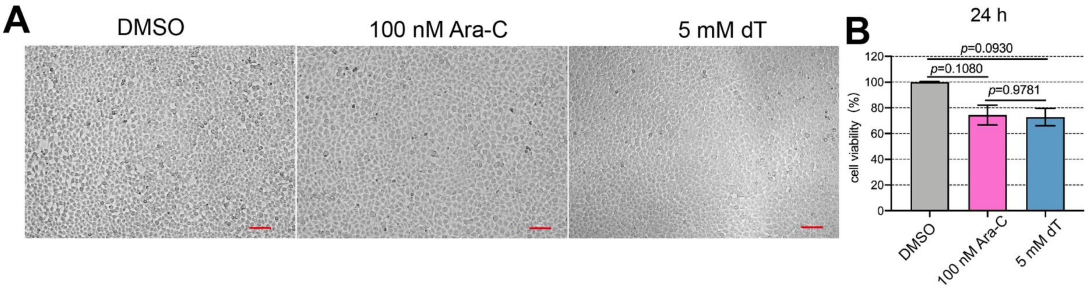

Figure. S8. Effects of DMSO, Arac-A, and dT on cell viability. A. Representative bright-field microscopy images of cells following 24-hour treatment with DMSO (control), Arac-A (100 nM), or dT (5 mM). Scale bar, 100 µm. **B.** Quantitative analysis of cell viability measured by CCK-8 assay after 24-hour treatment with the indicated compounds. Data are presented as the mean percentage of cell viability relative to the DMSO control group (set as 100%) ± s.d. from three independent experiments (n = 3).

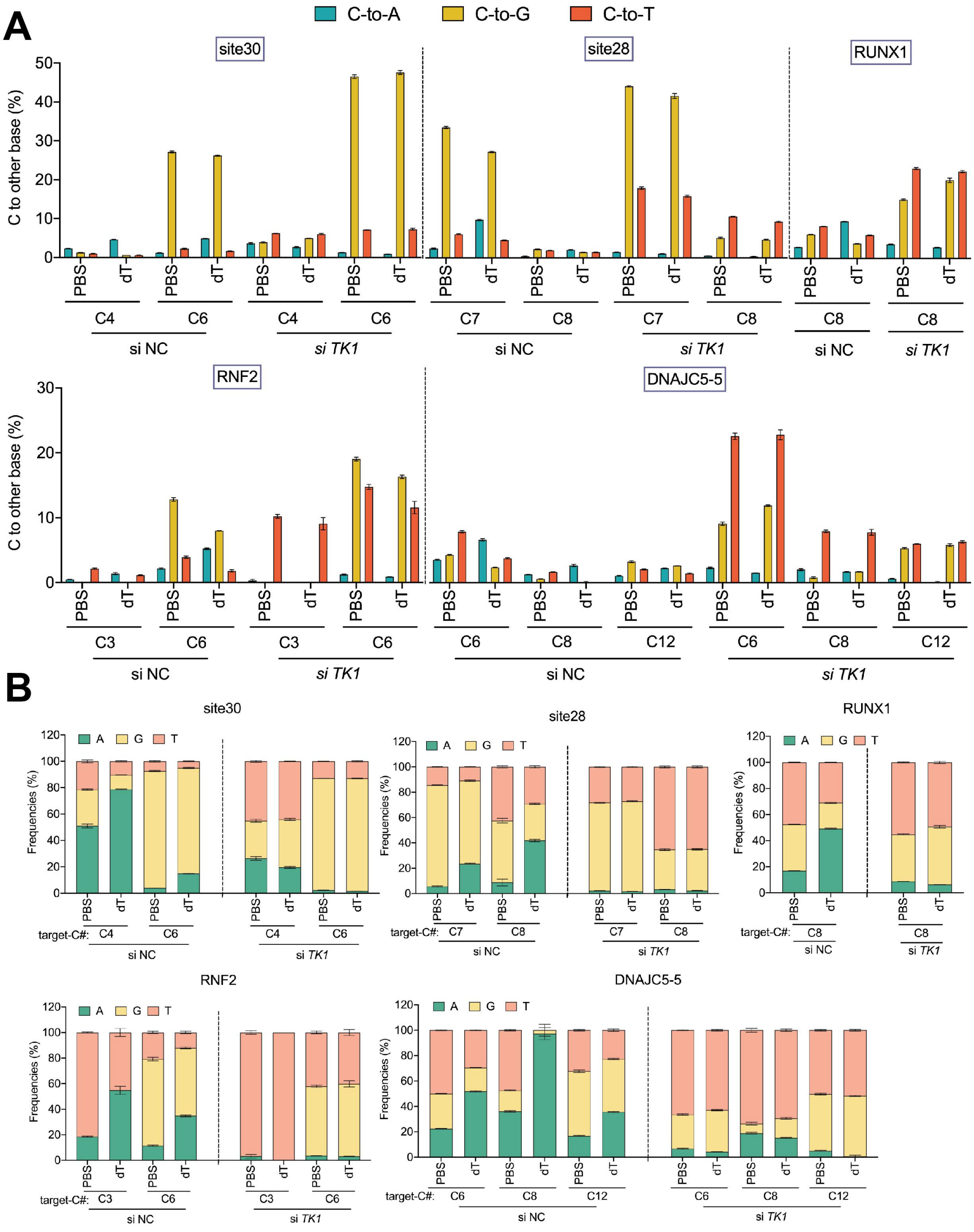

Figure. S9. Editing outcomes of CGBE with and without *TK1* depletion. A. Base editing efficiency mediated by CGBE under *TK1* knockdown (si-*TK1*) versus non-targeting control (si-NC). **B.** Product distribution of CGBE-mediated editing under *TK1* knockdown (si-*TK1*) versus non-targeting control (si-NC). The presented data are representative of three independent experiments, and error bars represent the standard deviation of the mean.

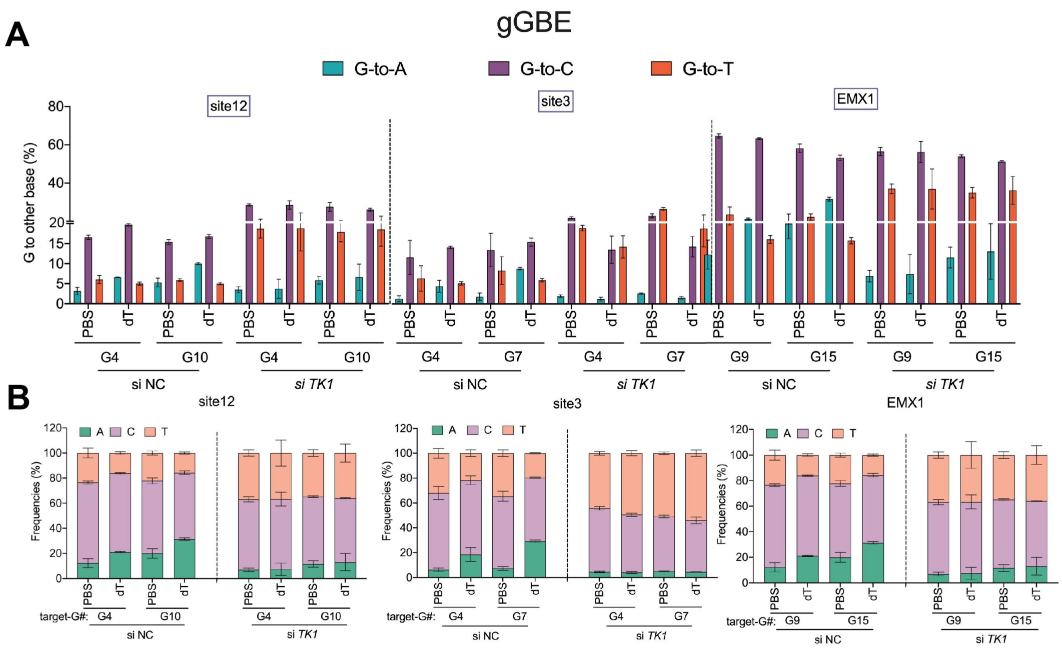

Figure. S10. Editing outcomes of gGBE with and without *TK1* depletion. A. Base editing efficiency mediated by gGBE under *TK1* knockdown (si-*TK1*) versus non-targeting control (si-NC). **B.** Product distribution of gGBE-mediated editing under *TK1* knockdown (si-*TK1*) versus non-targeting control (si-NC). The presented data are representative of three independent experiments, and error bars represent the standard deviation of the mean.

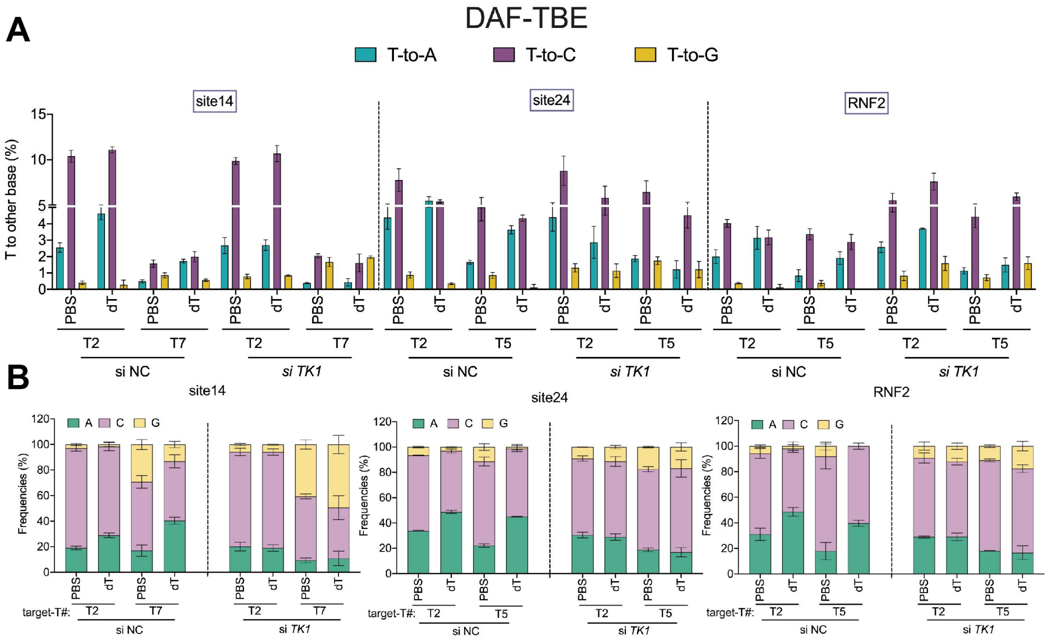

Figure. S11. Editing outcomes of DAF-TBE with and without *TK1* depletion. A. Base editing efficiency mediated by DAF-TBE under *TK1* knockdown (si-*TK1*) versus non-targeting control (si-NC). **B.** Product distribution of DAF-TBE-mediated editing under *TK1* knockdown (si-*TK1*) versus non-targeting control (si-NC). The presented data are representative of three independent experiments, and error bars represent the standard deviation of the mean.

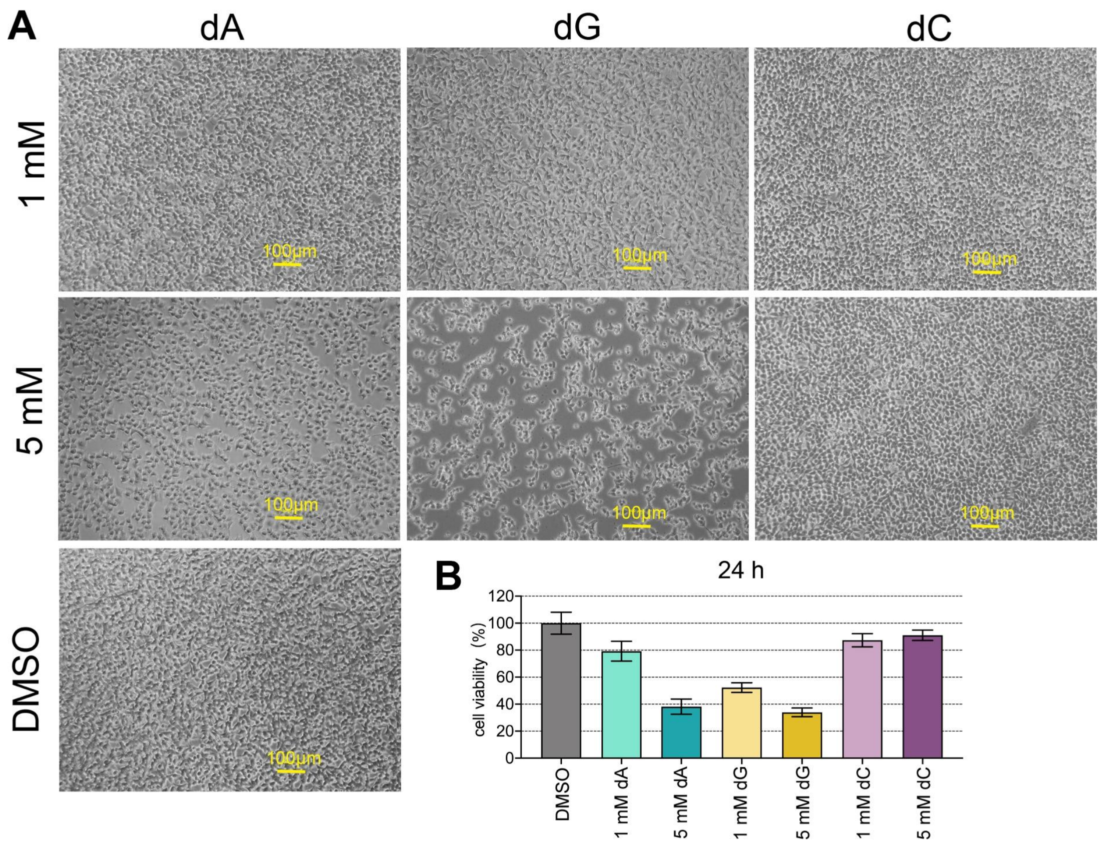

Figure. S12. Effects of deoxynucleoside (dN) on cell viability. A. Representative bright-field microscopy images of cells following 24-hour treatment with DMSO or dN. Scale bar, 100 µm. **B.** Quantitative analysis of cell viability measured by CCK-8 assay after 24-hour treatment with DMSO or dN. Data are presented as the mean percentage of cell viability relative to the DMSO control group (set as 100%) ± s.d. from three independent experiments (n = 3).

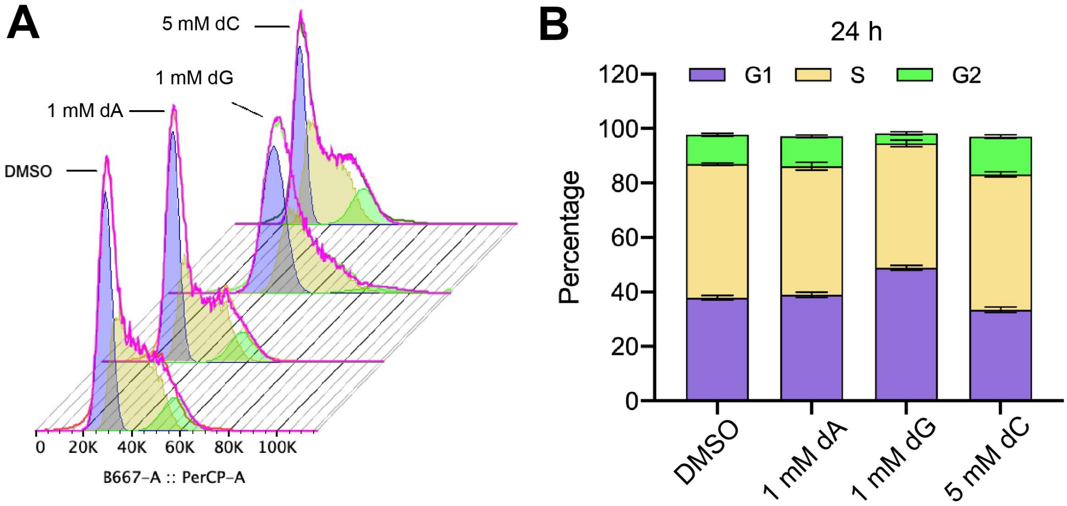

Figure. S13. Cell cycle analysis following 24-hour treatment with DMSO or dN. **A.** FlowJo-modeled cell cycle distributions. **B.** Quantitative phase allocation (see Methods).

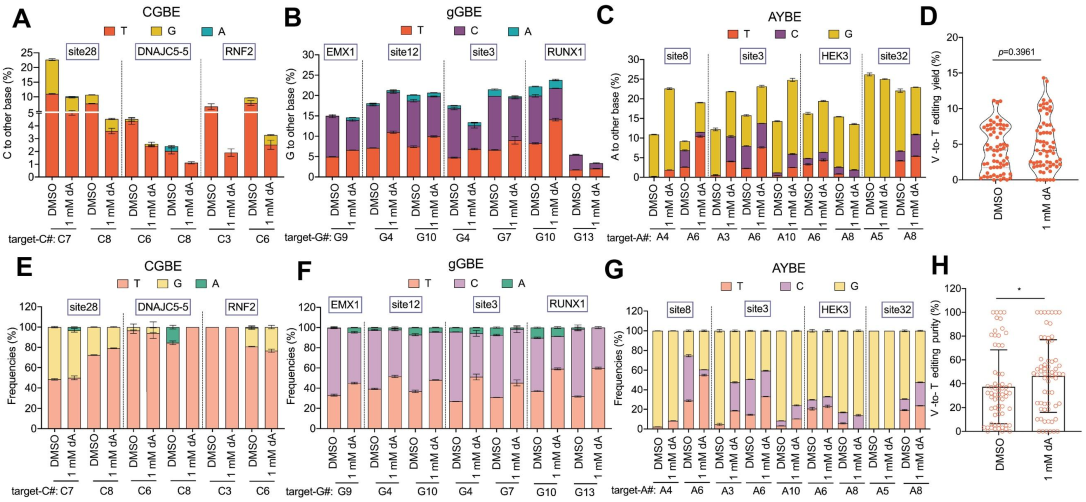

Figure. S14. Effects of 1mM dA on V-to-T editing outcomes at endogenous loci in HeLa cells. A. Endogenous locus editing efficiency following treatment with PBS or 1 mM dA. Data represent mean ± s.d. of three independent experiments. **B.** V-to-T editing efficiencies across all replicates and target sites. Data for each editing outcome are pooled from all eleven endogenous loci and three biological replicates. **C.** Endogenous locus product distribution after treatment with PBS or 1mM dA. Data are shown as mean ± s.d. from three independent experiments. **D.** V-to-T editing purity across all replicates and target sites (* $p < 0.05$).

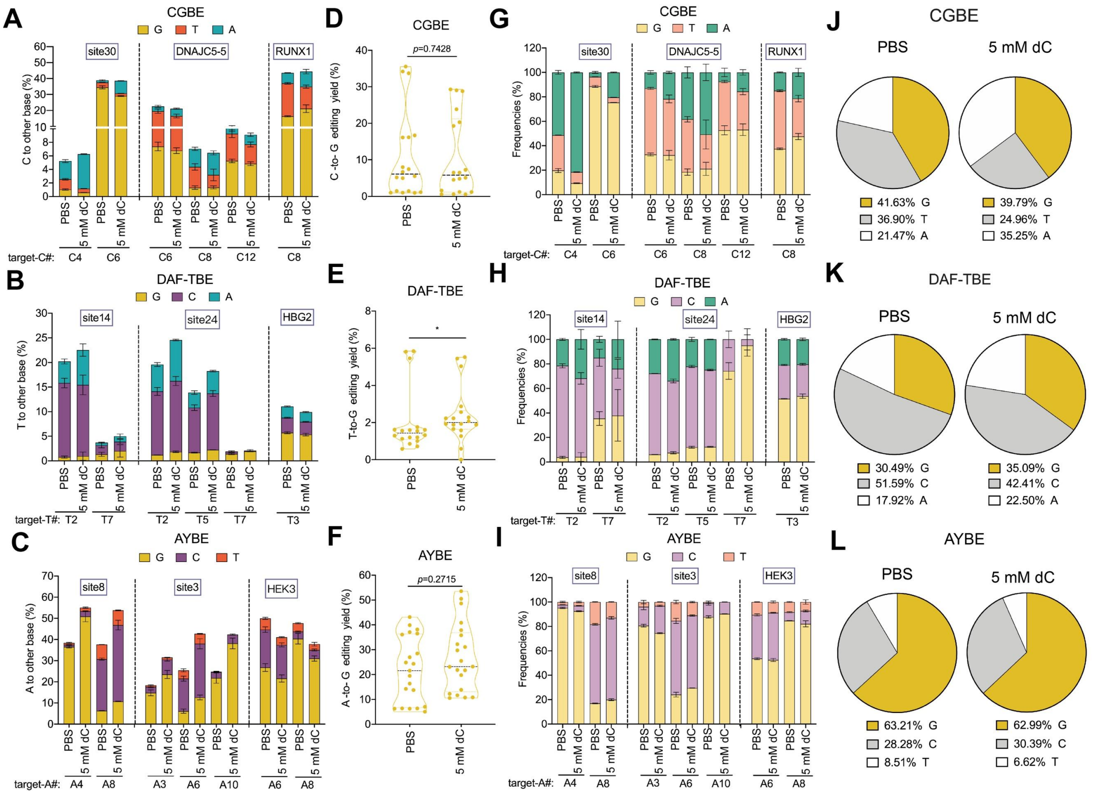

Figure S15. Effects of dC on H(C/T/A)-to-G conversions by glycosylase base editors. A. Endogenous locus editing efficiencies of CGBE with 5 mM dC in HEK293T cells. **B.** Editing efficiency of DAF-TBE. **C.** Editing efficiency of AYBE. **D.** C-to-G editing efficiency mediated by CGBE. **E.** T-to-G editing efficiency mediated by DAF-TBE (* $p < 0.05$). **F.** A-to-G editing efficiency mediated by AYBE (* $p < 0.05$). **G.** Product distribution of CGBE-mediated editing. **H.** Product distribution of gGBE-mediated editing. **I.** Product distribution of AYBE-mediated editing. **J.** Composition of editing products resulting from CGBE across three genomic loci. **K.** Composition of editing products resulting from DAF-TBE across three genomic loci. **L.** Composition of editing products resulting from AYBE across three genomic loci.

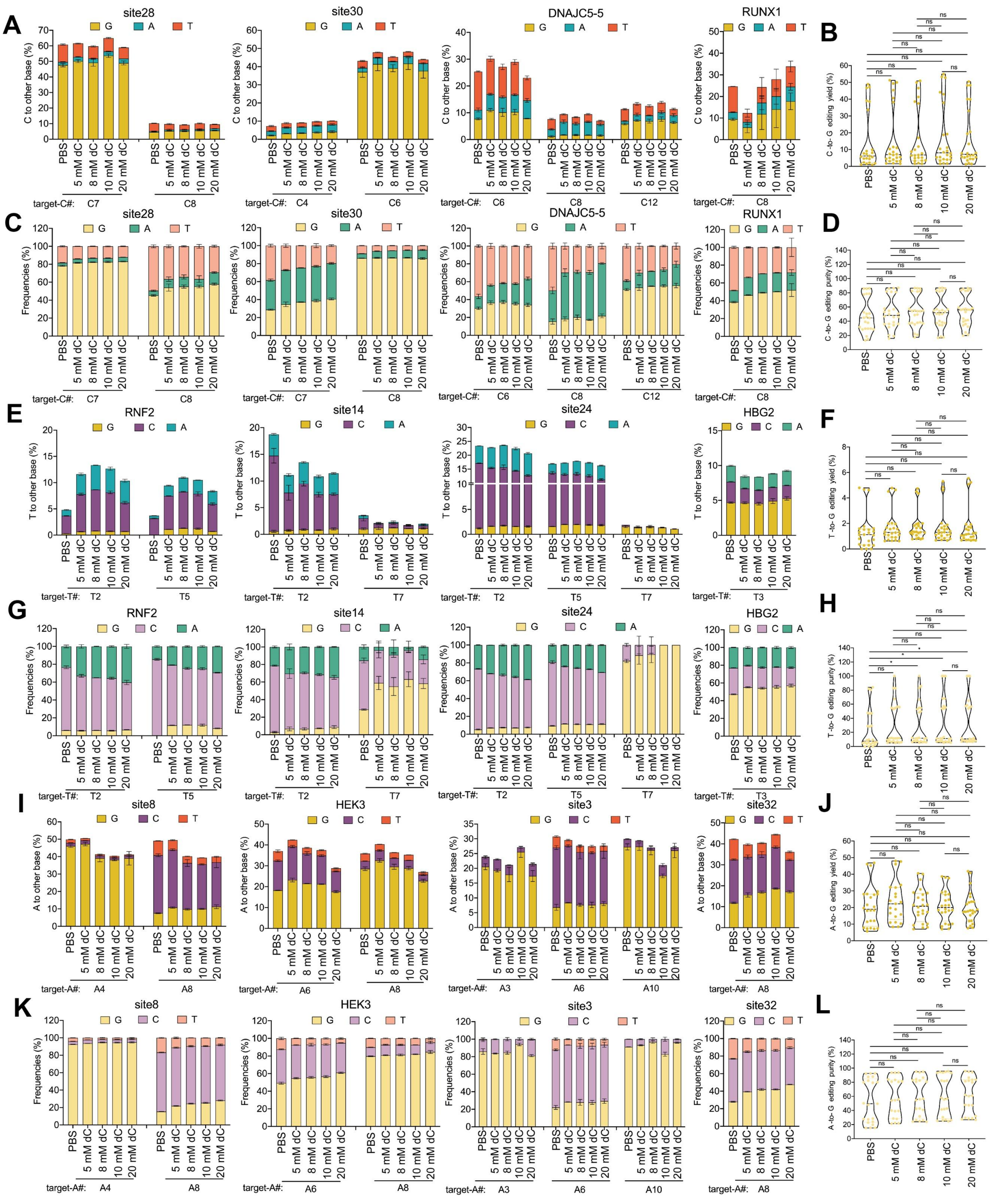

Figure S16. Effects of graded dC concentrations on the efficiency and purity of CGBE-mediated editing at endogenous loci in HEK293T. A. Endogenous locus editing efficiencies of CGBE with graded dC concentrations in HEK293T cells. Data represent mean ± s.d. (n = 3). **B.** The C-to-G editing efficiencies across all replicates and target sites. Data combined from four endogenous loci across three biological replicates (ns = not significant). **C.** Product distribution of CGBE-mediated editing with different dC concentrations. Data are shown as mean ± s.d. (n = 3). **D.** The C-to-G editing purity across all replicates and target sites (ns = not significant). **E.**

Effects of graded dC concentrations on DAF-TBE editing at endogenous loci in HEK293T cells. Data represent mean ± s.d. (n = 3). **F.** The T-to-G editing efficiencies across all replicates and target sites. Data pooled from four loci and three replicates (ns = not significant). **G.** Product distribution of DAF-TBE editing across dC concentrations. Data are shown as mean ± s.d. (n = 3). **H.** The T-to-G editing purity across all replicates and target sites (* $p < 0.05$, ns = not significant). **I.** AYBE editing efficiencies with graded dC. Data represent mean ± s.d. (n = 3). **J.** The A-to-G editing efficiencies across all replicates and target sites. Data pooled from four loci and three replicates (ns = not significant). **K.** Impact of dC concentration on the product profile of AYBE editing. Data are shown as mean ± s.d. (n = 3). **L.** The A-to-G editing purity across all replicates and target sites (ns = not significant).

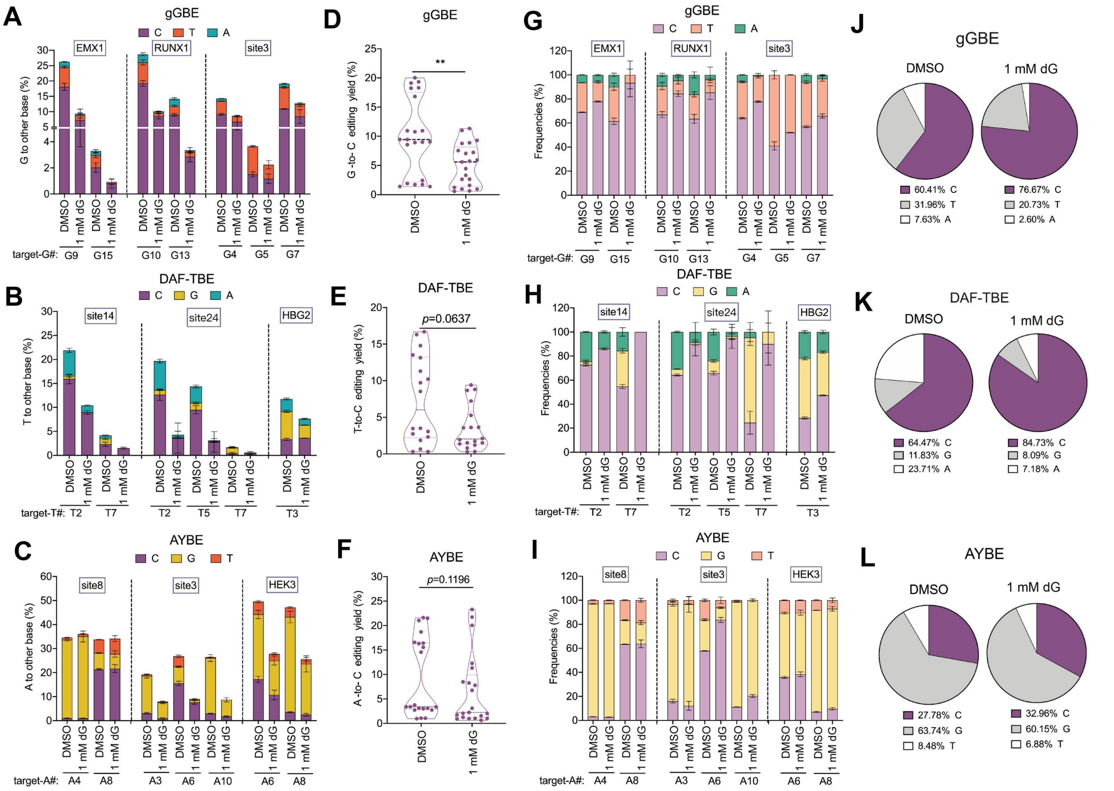

Figure S17. Effects of dG on D(G/T/A)-to-C conversion by glycosylase base editors. A. Endogenous locus editing efficiencies of gGBE with 1mM dG in HEK293T cells. Data are shown as mean ± s.d. (n = 3). **B.** Editing efficiency of DAF-TBE. Data are shown as mean ± s.d. (n = 3). **C.** Editing efficiency of AYBE. Data are shown as mean ± s.d. (n = 3). **D.** G-to-C editing efficiency mediated by gGBE (** $p < 0.01$). **E** T-to-C editing efficiency mediated by DAF-TBE (* $p < 0.05$). **F.** A-to-C editing efficiency mediated by AYBE. **G.** Product distribution of gGBE-mediated editing. Data are shown as mean ± s.d. (n = 3). **H.** Product distribution of DAF-TBE-mediated editing. Data are shown as mean ± s.d. (n = 3). **I.** Product distribution of AYBE-mediated editing. Data are shown as mean ± s.d. (n = 3). **J.** Composition of editing products resulting from gGBE across three genomic loci. **K.** Composition of editing products resulting from DAF-TBE across three genomic loci. **L.** Composition of editing products resulting from AYBE across three genomic loci.

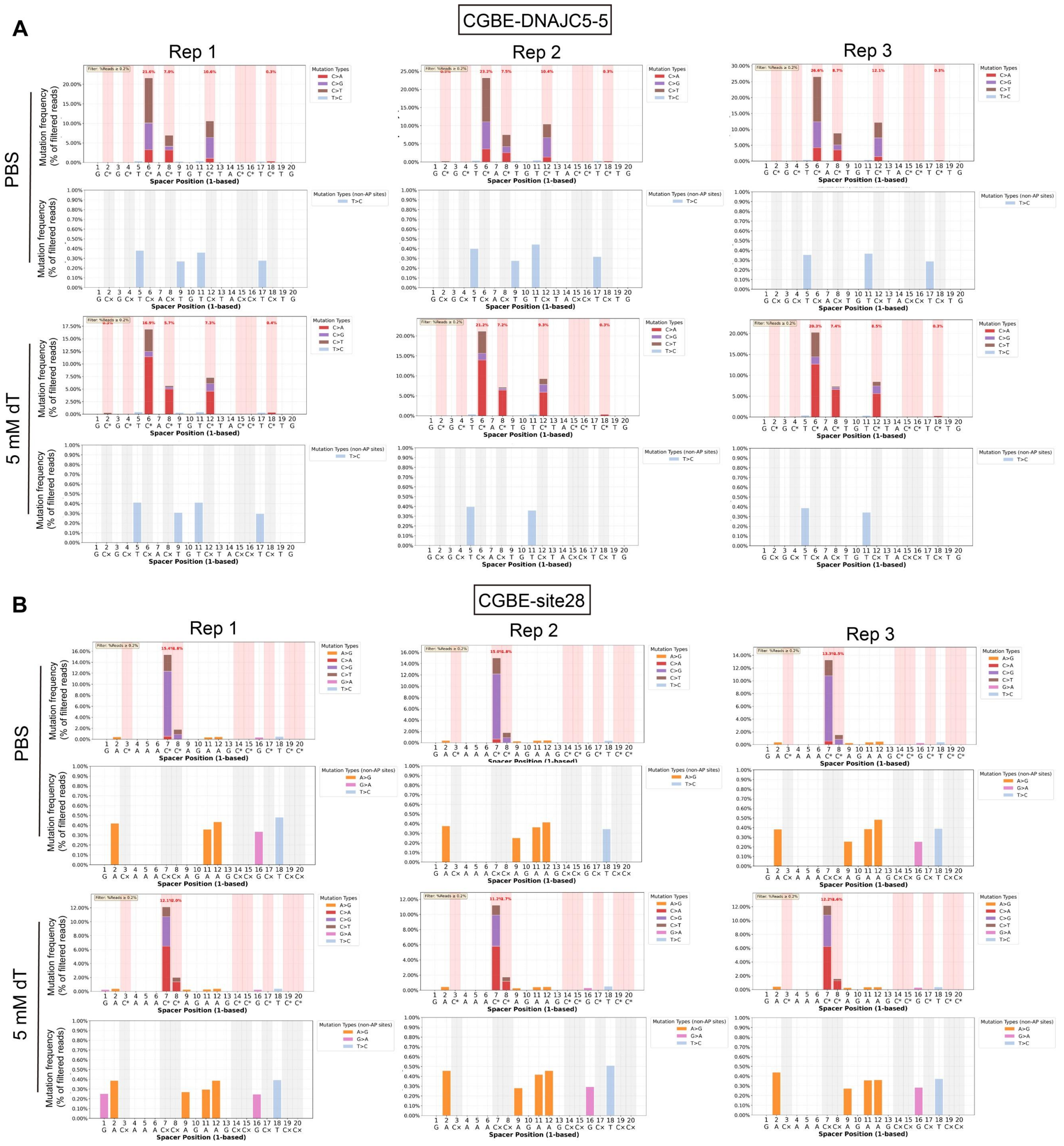

Figure S18. Deep sequencing analysis of edited loci and flanking regions in CGBE-mediated base editing.

A. Analysis of the *DNAJC5-5* locus. Experimental groups (PBS and 5 mM dT) are labeled on the left. For each group, the upper section displays editing outcomes at the target locus for three biological replicates (Rep1, Rep2, Rep3). Mutation frequency (y-axis) is plotted against spacer position (x-axis). Spacer nucleotides are numbered sequentially starting from position 1 (PAM - distal). All cytosines (C) within the spacer are highlighted in red. The total conversion efficiency for the target site is indicated above each sequence profile. The lower panel displays, in matched order, the sequencing results for the flanking regions from the corresponding three replicates.

Cytosine positions corresponding to the spacer are shaded gray for reference. **B.** Analysis of the site28 locus, performed identically as described in A. All sequencing data shown are derived from experiments presented in Figure 2.

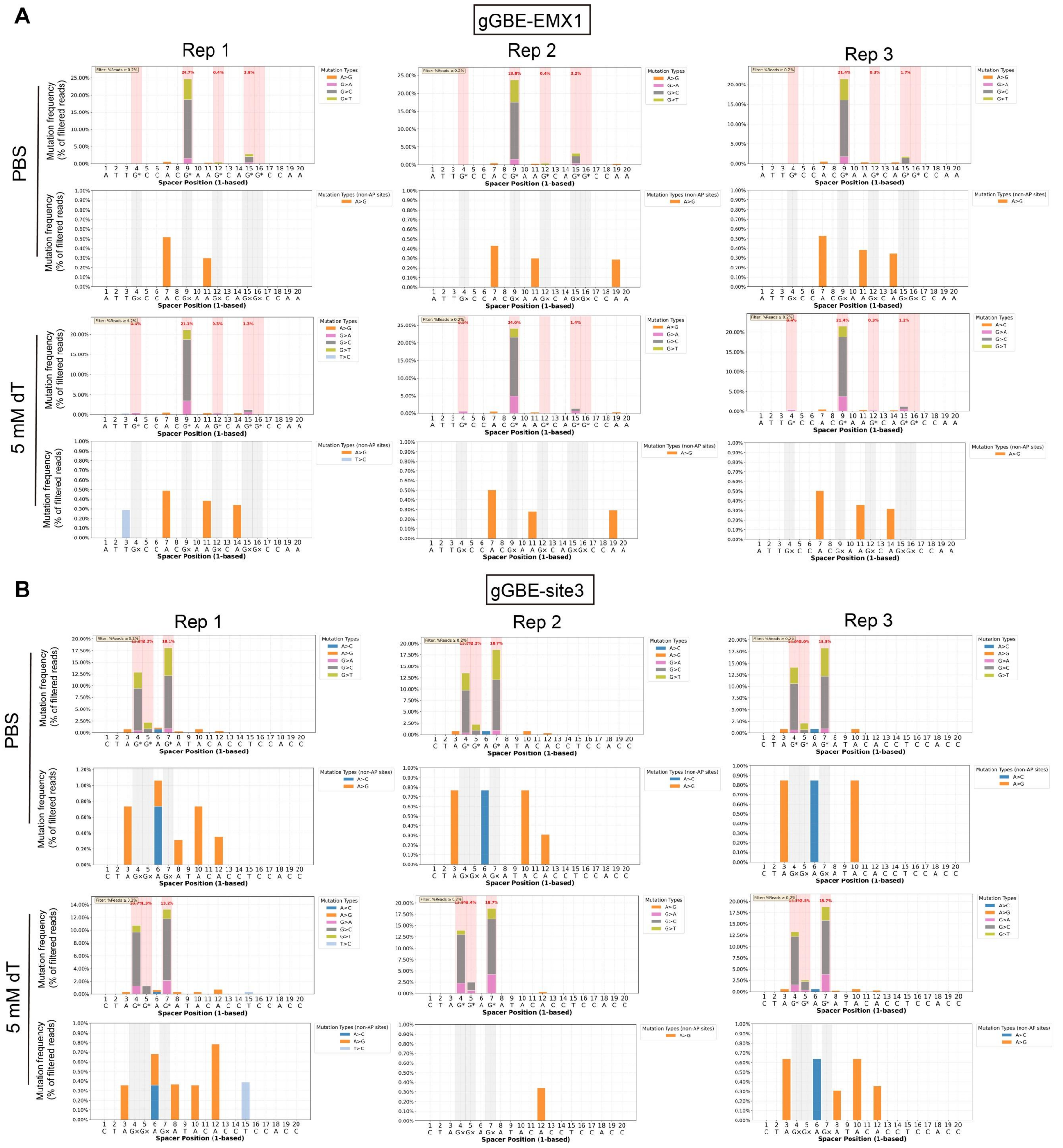

Figure S19. Deep sequencing analysis of edited loci and flanking regions in gGBE-mediated base editing. A. Analysis of the *EMX1* locus. Experimental groups (PBS and 5 mM dT) are indicated on the left. For each group, the upper panel shows editing outcomes at the target locus across three biological replicates (Rep1, Rep2, Rep3). Mutation frequency (y-axis) is plotted against spacer position (x-axis). Spacer nucleotides are numbered sequentially from position 1 (PAM - distal). All guanines (G) within the spacer are highlighted in red. The overall conversion efficiency is summarized above each profile. The lower panel displays, in matched order, sequencing results for the flanking regions of the same three replicates. Guanine positions corresponding to the spacer are shaded gray for reference. **B.** Analysis of the

site3 locus, performed identically as described in A. All sequencing data are derived from experiments presented in Figure 3.

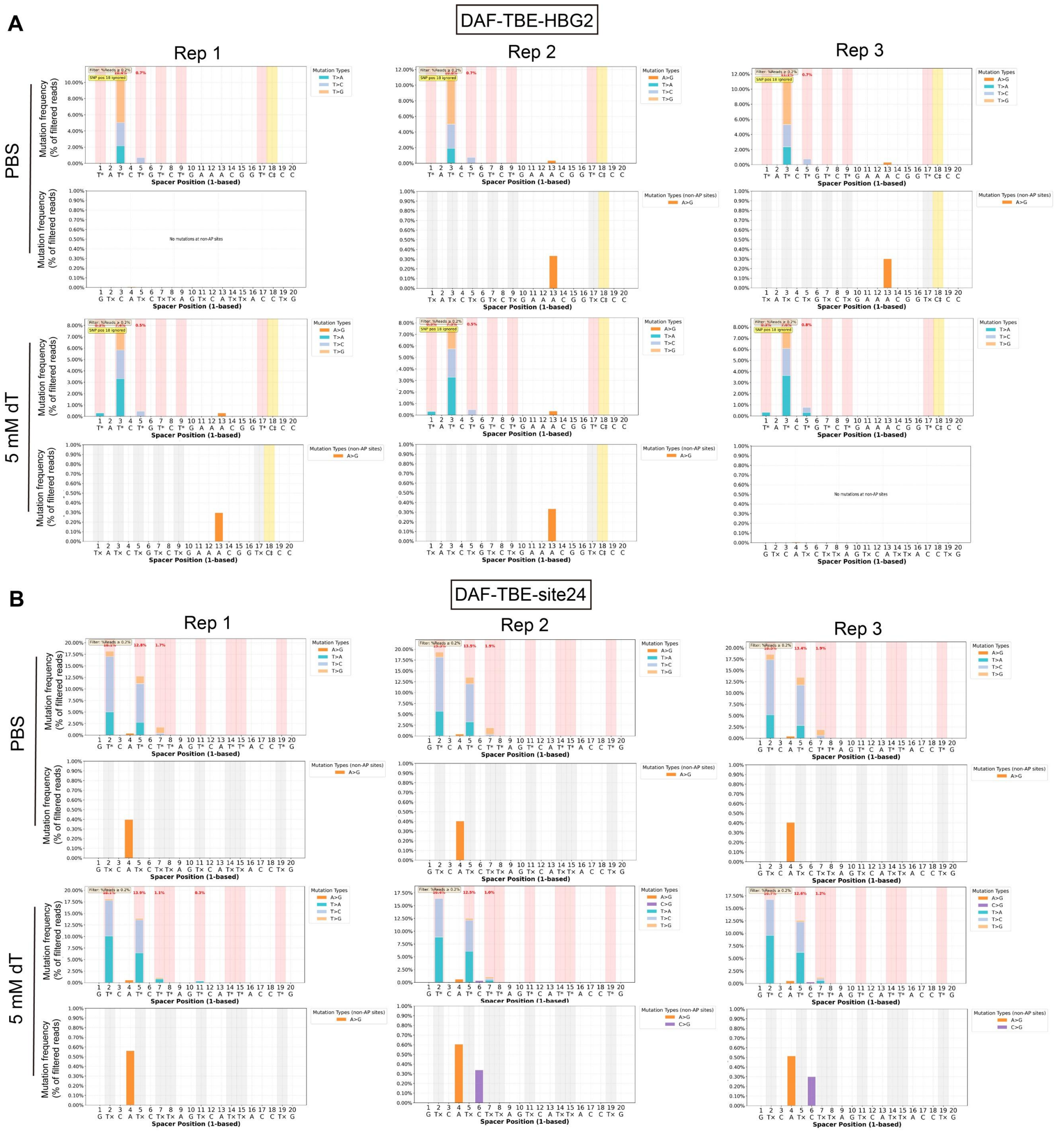

Figure S20. Deep sequencing analysis of edited loci and flanking regions in DAF-TBE-mediated base editing. A. Analysis of the *HBG2* locus. Experimental groups (PBS and 5 mM dT) are indicated. For each group, the upper panel shows target - locus editing outcomes across three biological replicates (Rep1–Rep3). Mutation frequency (y - axis) is plotted against spacer position (x - axis); positions are numbered from 1 (PAM - distal). All thymines (T) within the spacer are highlighted in red. Total conversion efficiency is shown above each profile. The single nucleotide polymorphism (SNP) at position 18 (C/T) is highlighted in yellow. The lower panel displays corresponding flanking - region sequencing results for the same three replicates. Spacer - corresponding thymine positions are shaded gray. **B.**

Analysis of the site24 locus, performed identically as in A. All data derive from experiments presented in Figure 3.

Tables S1. List of the targets tested in this study.

sgRNA	Target sequence **(PAM)**	Oligo-F	Oligo-R
EGFP-reporter	CCGTCGGTCAGGGTGGTCACGA **GGG**	CACCGCCGTCGGTCAGGGTGGTCACGA	AAACTCGTGACCACCCTGACCGACGGC
Site30	GAACACAAAGCATAGACTGC **GGG**	CACCGAACACAAAGCATAGACTGC	AAACGCAGTCTATGCTTTGTGTTC
Site28	GACAAACCAGAAGCCGCTCC **TGG**	CACCGACAAACCAGAAGCCGCTCC	AAACGGAGCGGCTTCTGGTTTGTC
DNAJC5-5	GCGCTCACTGTCTACCTCTG **GGG**	CACCGCGCTCACTGTCTACCTCTG	AAACCAGAGGTAGACAGTGAGCGC
RUNX1	GCATTTTCAGGAGGAAGCGA **TGG**	CACCGCATTTTCAGGAGGAAGCGA	AAACTCGCTTCCTCCTGAAAATGC
EMX1	ATTGCCACGAAGCAGGCCAA **TGG**	CACCGATTGCCACGAAGCAGGCCAA	AAACTTGGCCTGCTTCGTGGCAATC
Site12	TCAGAAAGTGGTGGCTGGTG **TGG**	CACCGTCAGAAAGTGGTGGCTGGTG	AAACCACCAGCCACCACTTTCTGAC
Site3	CTAGGAGATACACCTCCACC **AGG**	CACCGCTAGGAGATACACCTCCACC	AAACGGTGGAGGTGTATCTCCTAGC

RNF2	GTCATCTTAGTCATTACCTG**AGG**	CACCGTCATCTTAGTCATTACCTG	AAACCAGGTAATGACTAAGATGAC
Site14	CTGGCCTGGGTCAATCCTTG**GGG**	CACCGCTGGCCTGGGTCAATCCTTG	AAACCAAGGATTGACCCAGGCCAGC
Site24	GTCATCTTAGTCATTACCTG**AGG**	CACCGTCATCTTAGTCATTACCTG	AAACCAGGTAATGACTAAGATGAC
HBG2	TATCTGTCTGAAACGGTCCC**TGG**	CACCGTATCTGTCTGAAACGGTCCC	AAACGGGACCGTTTCAGACAGATAC
Site8	GTCATCCAGTGCTACCGCTG**TGG**	CACCGTCATCCAGTGCTACCGCTG	AAACCAGCGGTAGCACTGGATGAC
HEK3	GGCCCAGACTGAGCACGTGA**TGG**	CACCGGCCCAGACTGAGCACGTGA	AAACTCACGTGCTCAGTCTGGGCC
